# Supplementary material for: Nylons with Highly-Bright and Ultralong Organic Room-Temperature Phosphorescence
Source: Nat Commun. 2024 May 23;15:4402. doi: 10.1038/s41467-024-48836-7 (PMC11116439; doi:10.1038/s41467-024-48836-7)
Supplement: Supplementary file 1 — Supplementary Information [file 41467_2024_48836_MOESM1_ESM.pdf]

Supplementary Information

Nylons with Highly-Bright and Ultralong Organic Room-Temperature Phosphorescence

Dian-Xue Ma,[1,2,3,4] Zhong-Qiu Li,[1,2,3] Kun Tang,[1,2,3] Zhong-Liang Gong,[1,2,3] Jiang-Yang Shao,[1,2,3] and Yu-Wu Zhong[1,2,3,4]*

[1]Key Laboratory of Photochemistry, Institute of Chemistry, Chinese Academy of Sciences, Beijing 100190, China.

[2]Beijing National Laboratory for Molecular Sciences, Beijing 100190, China.

[3]CAS Research/Education Center for Excellence in Molecular Sciences, Institute of Chemistry, Chinese Academy of Sciences, Beijing 100190, China.

[4]School of Chemical Sciences, University of Chinese Academy of Sciences, Beijing 100049, China.

*e-mail: zhongyuwu@iccas.ac.cn

Supplementary Methods

General information for synthesis. ^{1}H and ^{13}C nuclear magnetic resonance (NMR) spectra were recorded on Bruker ADVANCE III 300 MHz or 400 MHz spectrometer using tetramethylsilane (TMS) as the internal standard. High-resolution electron ionization mass spectra were collected on Waters GCT mass spectrometer.

Synthesis of 2-phenyl-1H-4,7-dicyanobenzo[d]imidazole (1)

A mixture of 2,3-diaminoterephthalonitrile (158.2 mg, 1.0 mmol), benzaldehyde (305.3 mg, 2.5 mmol) and sodium metabisulfite (380.1 mg, 2.0 mmol) in 5 mL of DMSO were heated at 140 ℃ for 24 h. After the completion of the reaction, the reaction mixture was cooled to room temperature and the solvent was removed under vacuum. The crude product was purified by column chromatography on silica gel (200–300 mesh) with petroleum/ethyl acetate (v/v, 5/1) as the eluent to give 214.7 mg of **1** as a light yellow solid in 88% yield. ^{1}H NMR (400 MHz, THF-d8): δ 13.08 (s, 1H), 8.30 (d, *J* = 3.7 Hz, 2H), 7.68 (s, 2H), 7.61 – 7.43 (m, 3H). ^{13}C NMR (100 MHz, THF-d8): δ 157.04, 146.77, 137.49, 132.11, 129.78, 128.57, 127.40, 127.14, 116.17, 115.94, 107.86, 99.93, 67.83, 67.61, 67.40, 67.18, 66.96, 25.71, 25.51, 25.31, 25.11, 24.91. HRMS (EI) for $C_{15}H_8N_4$ $[M+H]^+$: calcd 244.0749, found 244.0744. Anal. Calcd for $C_{15}H_8N_4$: C, 73.76; H, 3.30; N, 22.94. Found: C, 73.66; H, 3.29; N, 22.78.

Synthesis of 2-phenyl-1H-5,6-dicyanobenzo[d]imidazole (2)

A mixture of 4,5-diaminophthalonitrile (237.2 mg, 1.5 mmol), benzaldehyde (458.0 mg, 3.75 mmol) and sodium metabisulfite (570.3 mg, 3.0 mmol) in 7 mL of DMSO were heated at 140 ℃ for 24 h. After the completion of the reaction, the reaction mixture was cooled to room temperature and the solvent was removed under vacuum. The crude product was purified by column chromatography on silica gel (200–300 mesh) with petroleum/ethyl acetate (v/v, 1/1) as the eluent to give 107.5 mg of **2** as a white solid in 29% yield. ^{1}H NMR (400 MHz, THF-d8): δ 12.77 (s, 1H), 8.26 (s, 1H), 8.22 - 8.15 (m, 2H), 8.06 (s, 1H), 7.58 – 7.50 (m, 3H). ^{13}C NMR (400 MHz, THF-d8): δ 158.35, 147.61, 138.38, 132.21, 130.04, 129.98, 128.26, 126.56, 118.39, 117.33, 109.48, 109.24, 67.93, 67.72, 67.50, 67.28, 67.06, 25.81, 25.61, 25.41, 25.21, 25.01. HRMS (EI) for $C_{15}H_8N_4$ $[M+H]^+$: calcd 244.0749, found 244.0743. Anal. Calcd for $C_{15}H_8N_4$: C, 73.76; H,

3.30; N, 22.94. Found: C, 73.43; H, 3.29; N, 22.36.

Synthesis of 2-methyl-1H-4,7-dicyanobenzo[d]imidazole (3)

A mixture of 2,3-diaminophthalonitrile (79.1 mg, 0.5 mmol), acetaldehyde (55.1 mg, 1.25 mmol) and sodium metabisulfite (190.1 mg, 1.0 mmol) in 5 mL of DMSO were heated at 140℃ for 24 h. After the completion of the reaction, the reaction mixture was cooled to room temperature and the solvent was removed under vacuum. The crude product was purified by column chromatography on silica gel (200–300 mesh) with petroleum/ethyl acetate (v/v, 1/1) as the eluent to give 52.5 mg of **2** as a white solid in 58% yield. ^{1}H NMR (400 MHz, THF-d8): δ 12.64 (s, 1H), 7.61 (s, 2H), 2.64 (s, 3H). ^{13}C NMR (400 MHz, THF-d8): δ 157.41, 146.45, 137.22, 126.35, 126.24, 116.17, 115.95, 107.24, 99.24, 67.83, 67.61, 67.40, 67.18, 66.96, 25.71, 25.51, 25.31, 25.11, 24.91, 14.88. HRMS (EI) for $C_{10}H_6N_4$ [M+H]$^+$: calcd 182.0592, found 182.0586. Anal. Calcd for $C_{10}H_6N_4$: C, 65.93; H, 3.32; N, 30.75. Found: C, 65.13; H, 3.30; N, 30.28.

Synthesis of 2-(4'-methoxy-[1,1'-biphenyl]-4-yl)-1H-4,7-dicyanobenzo[d]imidazole (4)

A mixture of 2,3-diaminophthalonitrile (158.2 mg, 1.0 mmol), 4-(4'-methoxyphenyl)-benzaldehyde (530.5 mg, 2.5 mmol) and sodium metabisulfite (380.1 mg, 2.0 mmol) in 7 mL of DMSO were heated at 140 ℃ for 24 h. After the completion of the reaction, the reaction mixture was cooled to room temperature and the solvent was removed under vacuum. The crude product was purified by column chromatography on silica gel (200–300 mesh) with dichloromethane/methanol (v/v, 100/1) as the eluent to give 184.7 mg of **4** as a white in 55% yield. ^{1}H NMR (400 MHz, THF-d8): δ 13.01 (s, 1H), 8.35 (d, *J* = 8.3 Hz, 2H), 7.79 (d, *J* = 8.3 Hz, 2H), 7.67 (d, *J* = 8.8 Hz, 4H), 7.01 (d, *J* = 8.7 Hz, 2H), 3.82 (s, 3H). ^{13}C NMR (400 MHz, THF-d8): δ 161.16, 156.95, 146.88, 144.56, 137.59, 132.87, 128.99, 128.83, 127.74, 127.57, 127.22, 127.05, 116.27, 115.98, 115.15, 107.64, 99.78, 67.83, 67.61, 67.39, 67.17, 66.96, 55.49, 25.71, 25.51, 25.31, 25.11, 24.91. HRMS (EI) for $C_{22}H_{14}N_4O$ [M+H]$^+$: calcd 350.1168, found 350.1157. Anal. Calcd for $C_{22}H_{14}N_4O$: C, 75.42; H, 4.02; N, 15.99. Found: C, 74.71; H, 4.02; N, 15.74.

Synthesis of 2-phenyl-1-methyl-4,7-dicyanobenzo[d]imidazole (6)

To a stirred solution of **1** (97.7 mg, 0.4 mmol) in 5 mL of dry DMF was added NaH (60% dispersion in mineral oil, 64 mg, 1.6 mmol) in two batches at 0 ℃. After stirring for 30 min at

0 ℃, iodomethane (113.6 mg, 0.8 mmol) was added and the mixture was stirred at rt for 5 h. The reaction mixture was poured into H_2O (10 mL) and extracted with ethyl acetate (2 × 30 mL). The combined organic phase was dried over Na_2SO_4. After filtration and evaporation of the solvents under reduced pressure, the crude product was purified by column chromatography on silica gel (petroleum ether/ethyl acetate 1/1) to yield 77.4 mg of **6** in 81% yield as a white solid. ^{1}H NMR (400 MHz, THF-d8): δ 7.95 - 7.81 (m, 2H), 7.73 (q, *J* = 7.9 Hz, 2H), 7.63-7.49 (m, 3H), 4.20 (s, 3H). ^{13}C NMR (400 MHz, THF-d8): δ 158.97, 145.88, 137.35, 131.55, 130.85, 129.75, 129.5, 128.43, 127.06, 116.36, 116.01, 108.40, 99.97, 67.83, 67.61, 67.39, 67.17, 66.96, 33.83, 25.71, 25.51, 25.31, 25.11, 24.91. HRMS (EI) for $C_{16}H_{10}N_4$ $[M+H]^+$: calcd 258.0509, found 257.0823. Anal. Calcd for $C_{16}H_{10}N_4$: C, 74.40; H, 3.90; N, 21.69. Found: C, 74.02; H, 3.85; N, 21.88.

Supplementary Figures

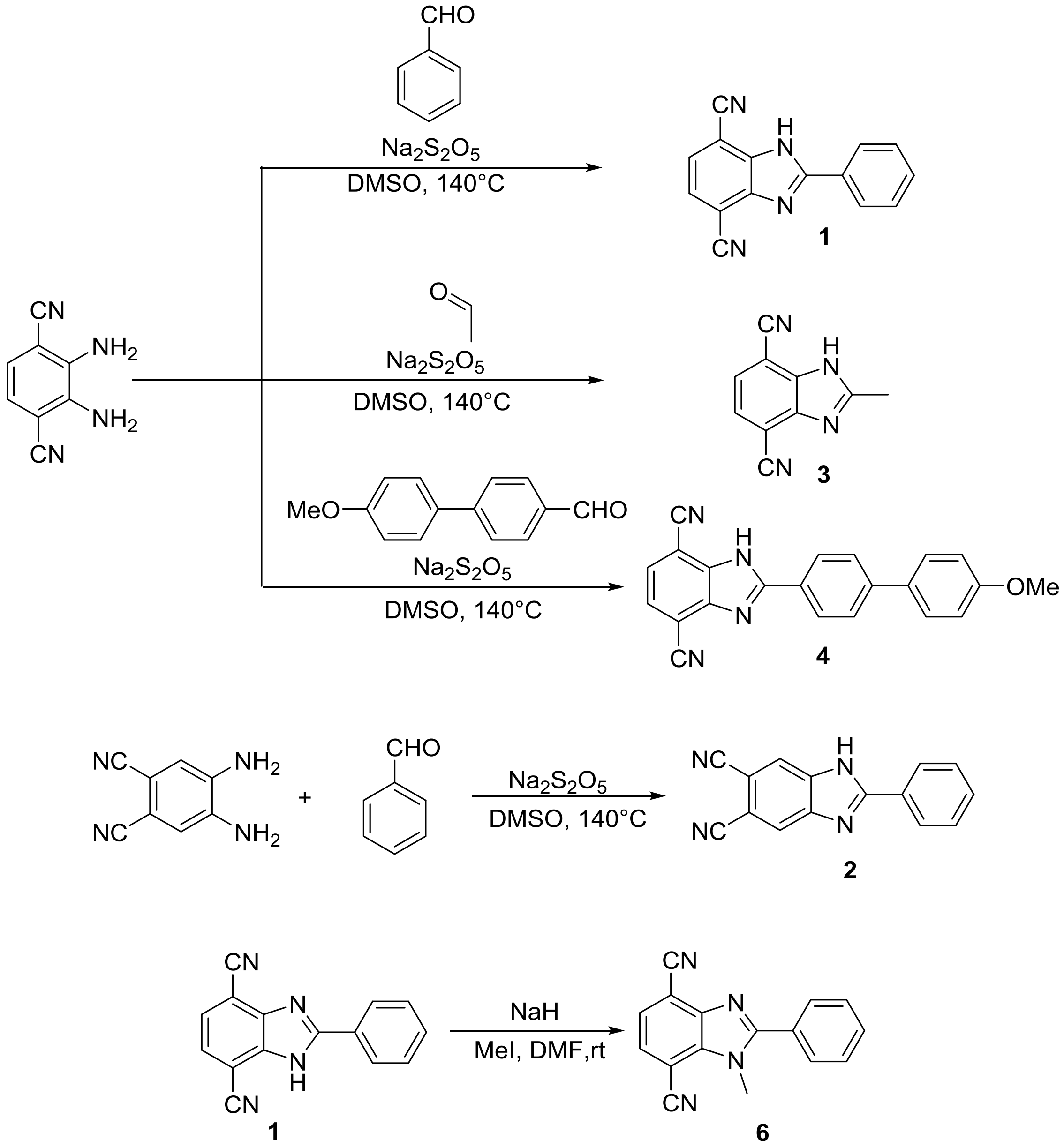

Supplementary Figure 1. Synthetic routes of the target molecules. The details for the synthetic routes of phosphors **1**, **2**, **3**, **4** and **6** were listed.

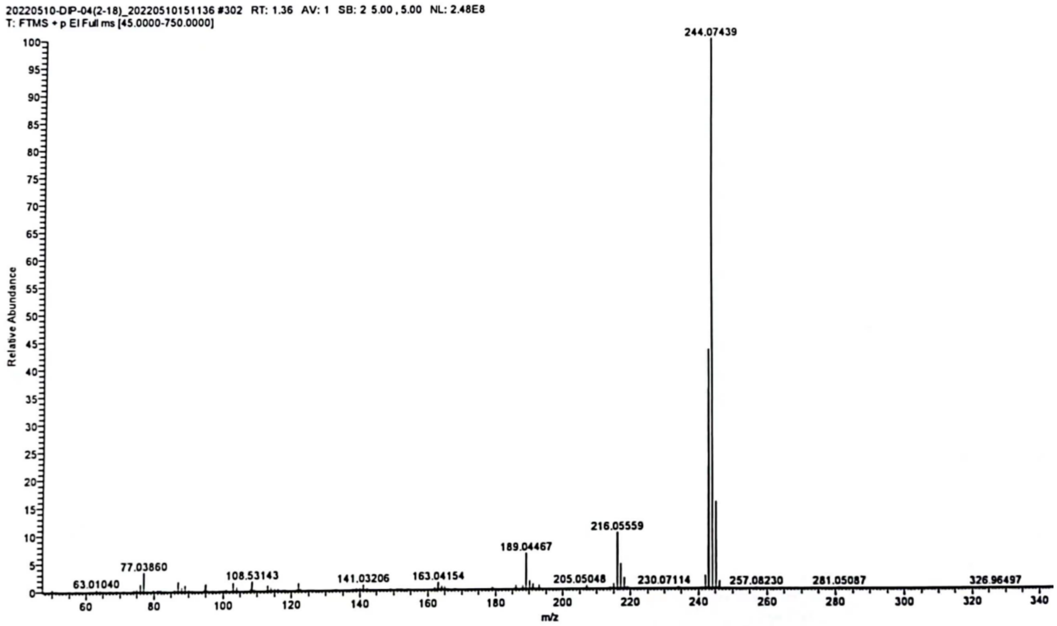

Supplementary Figure 2. HRMS spectrum of **1**.

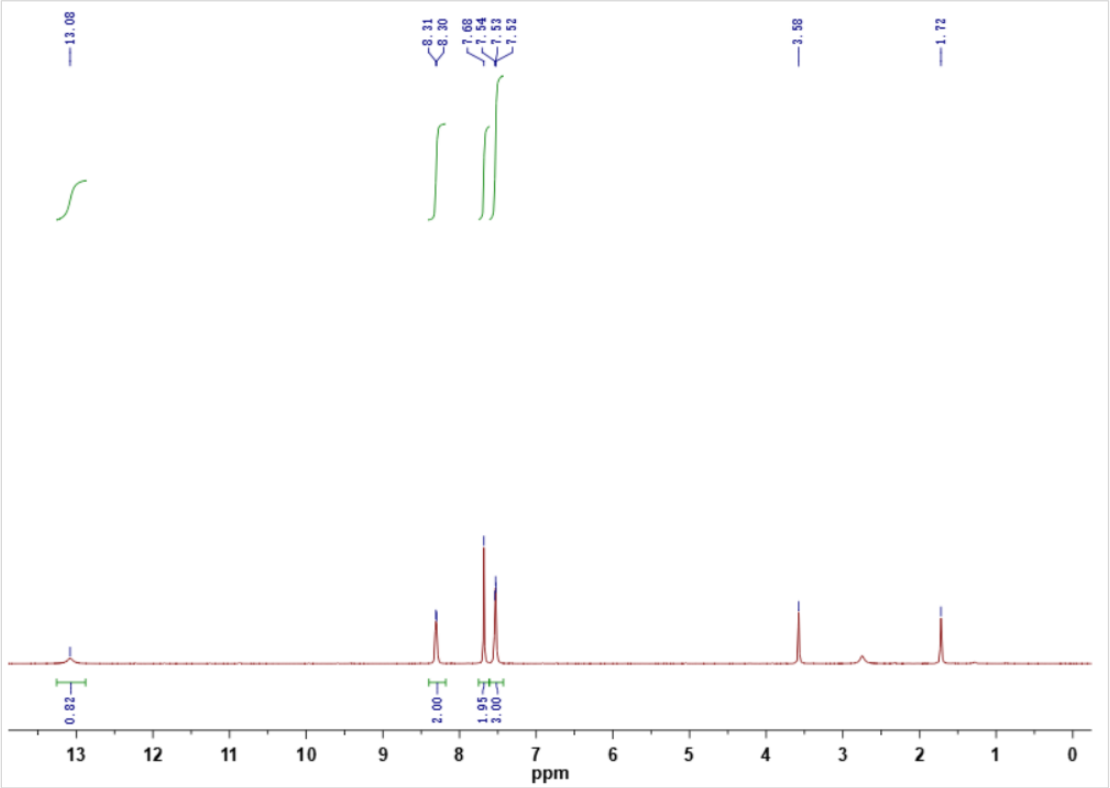

Supplementary Figure 3. ^{1}H NMR spectrum of **1** in THF-d8.

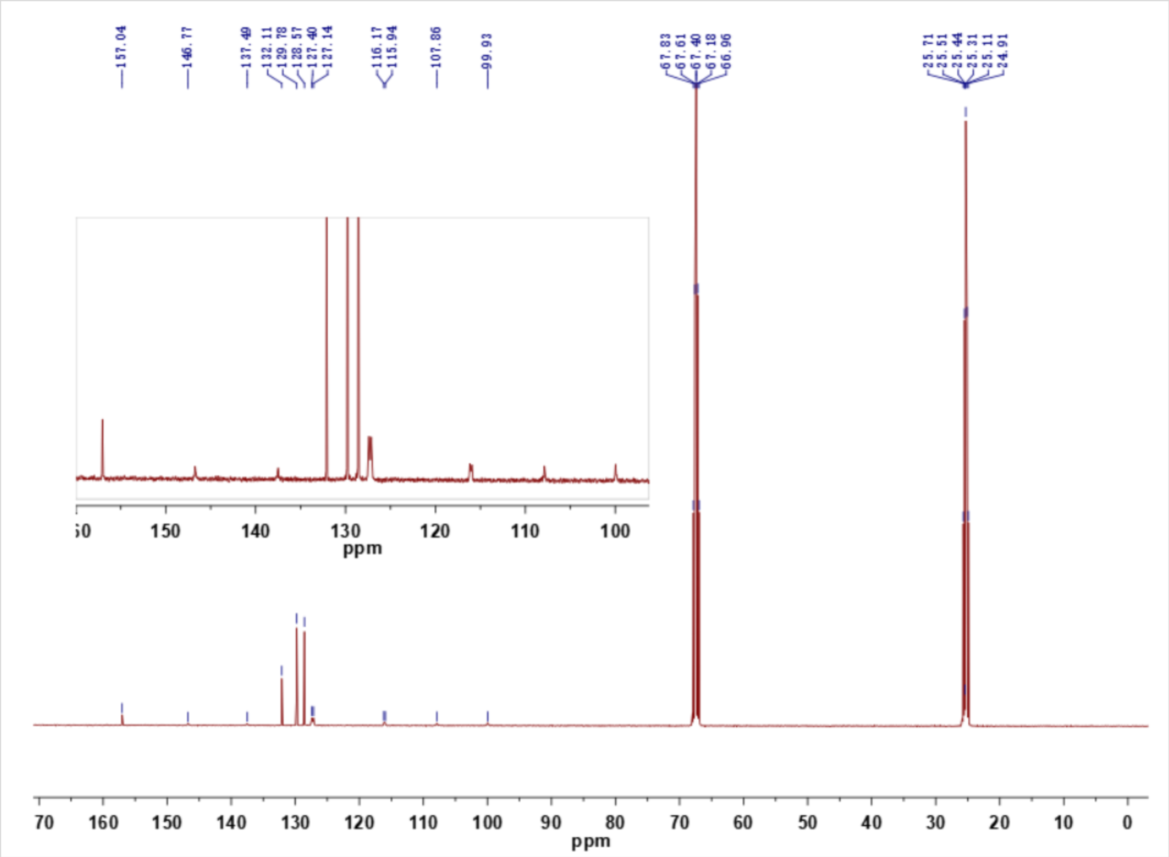

Supplementary Figure 4. ^{13}C NMR spectrum of **1** in THF-d8.

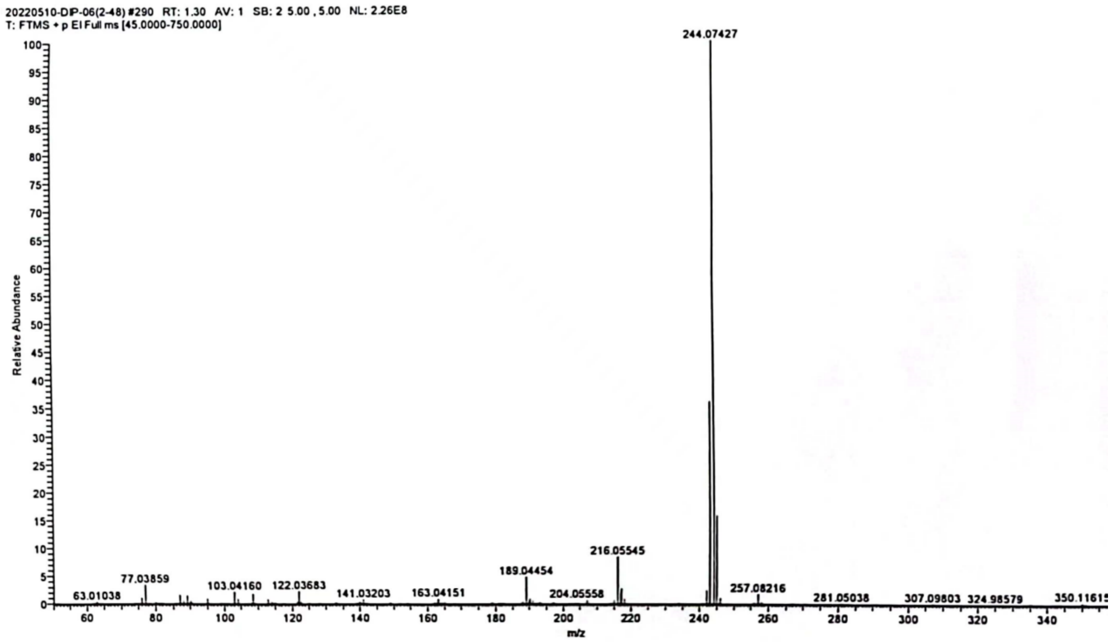

Supplementary Figure 5. HRMS spectrum of **2**.

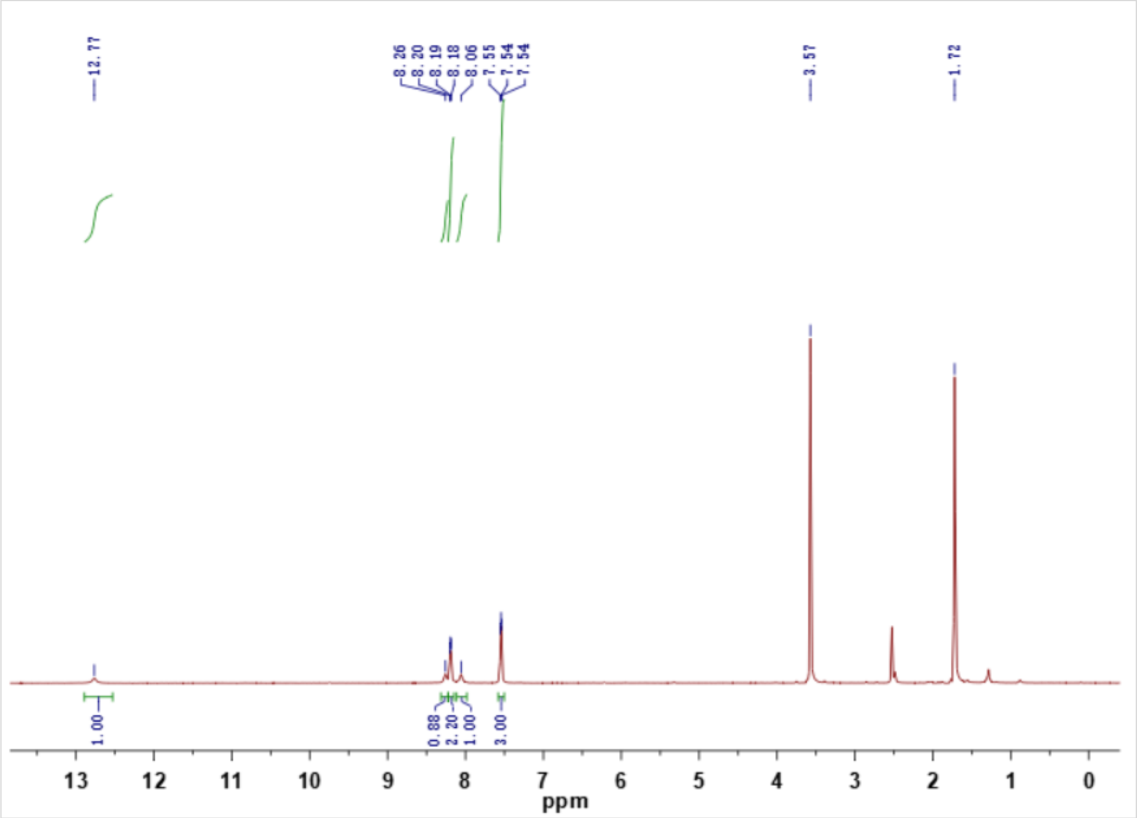

Supplementary Figure 6. ^{1}H NMR spectrum of **2** in THF-d8.

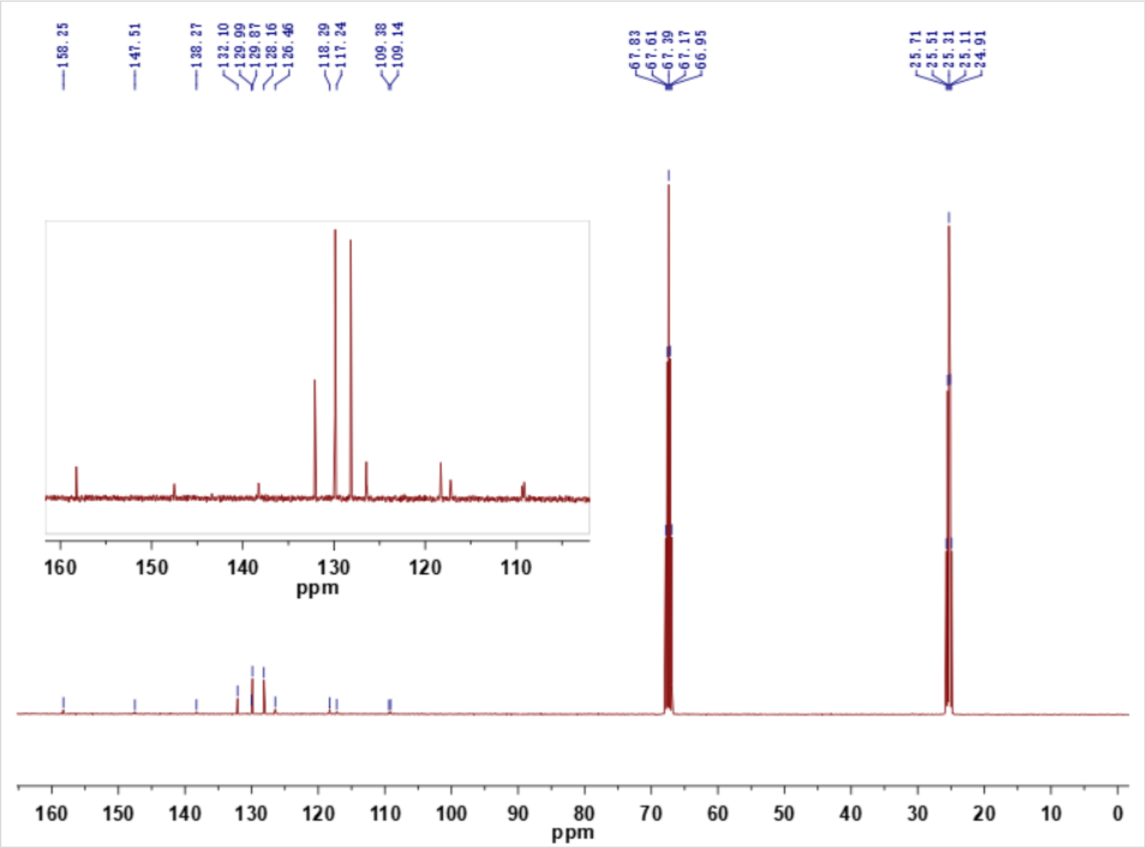

Supplementary Figure 7. ^{13}C NMR spectrum of **2** in THF-d8.

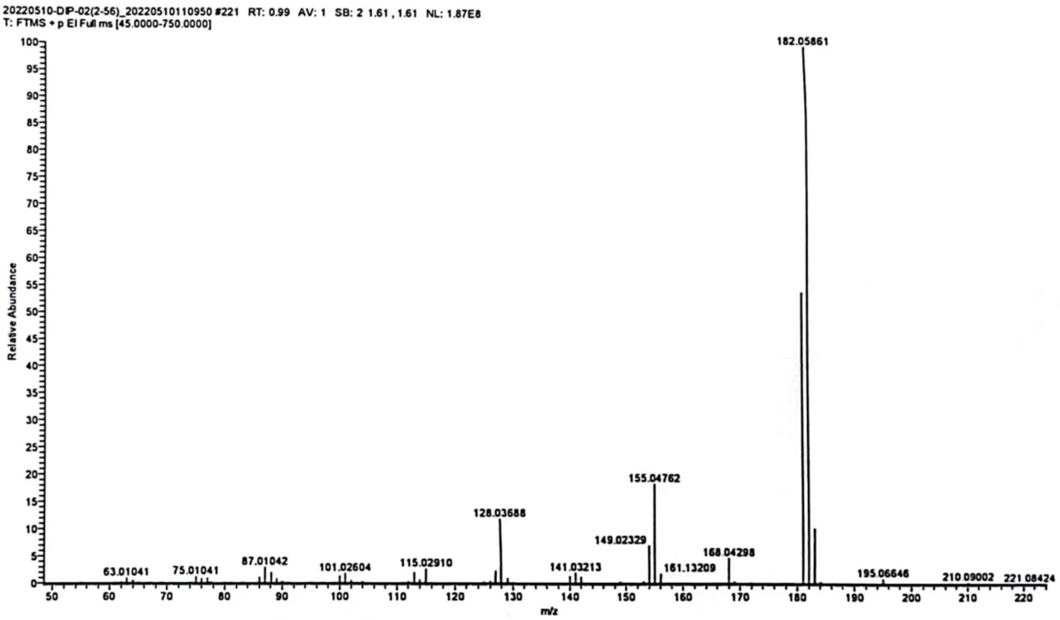

Supplementary Figure 8. HRMS spectrum of **3**.

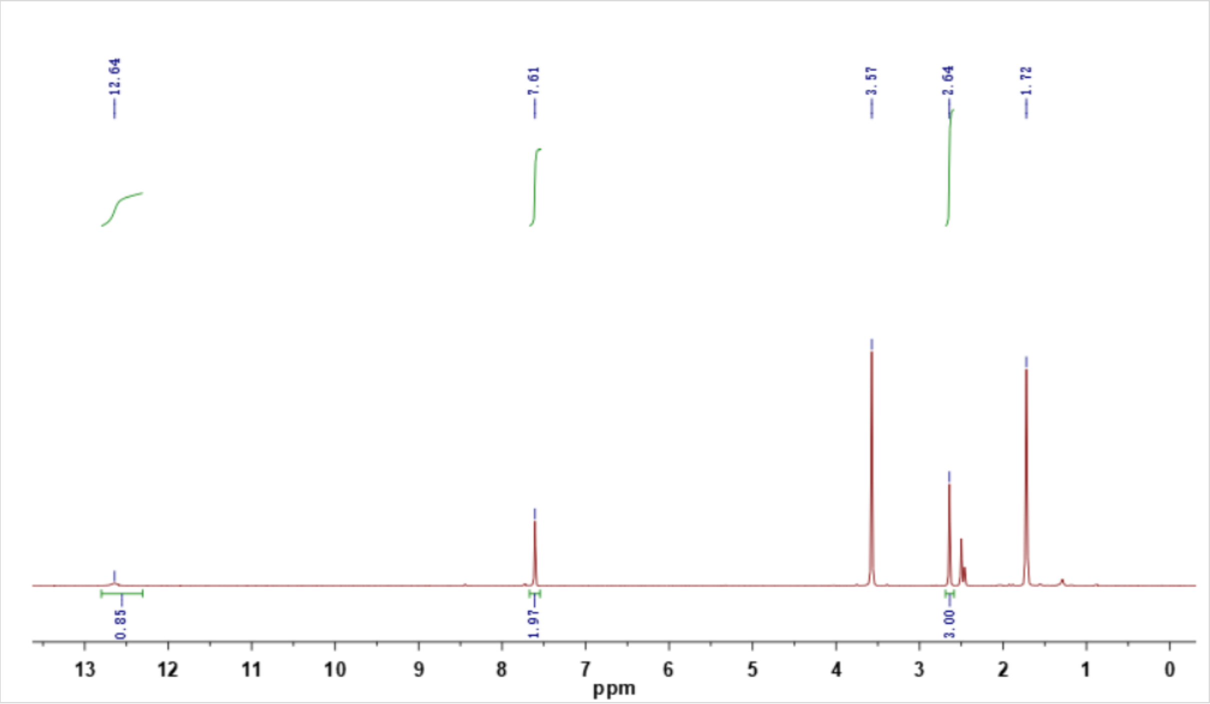

Supplementary Figure 9. ^{1}H NMR spectrum of **3** in THF-d8.

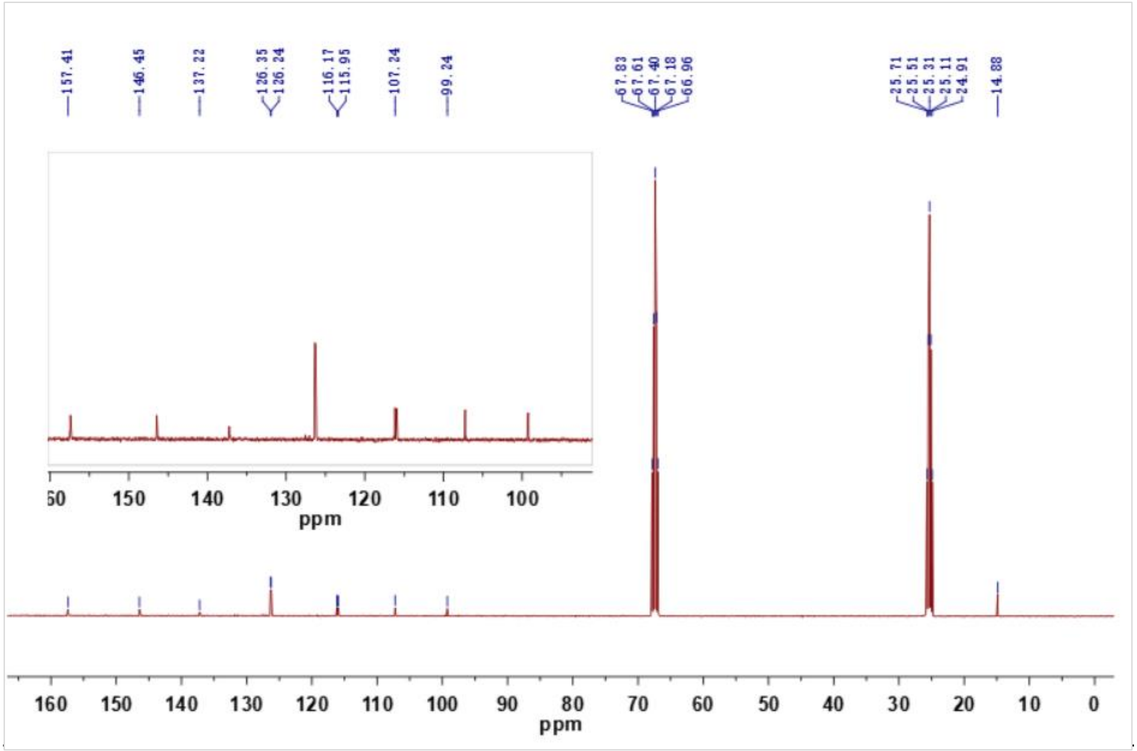

Supplementary Figure 10. ^{13}C NMR spectrum of **3** in THF-d8.

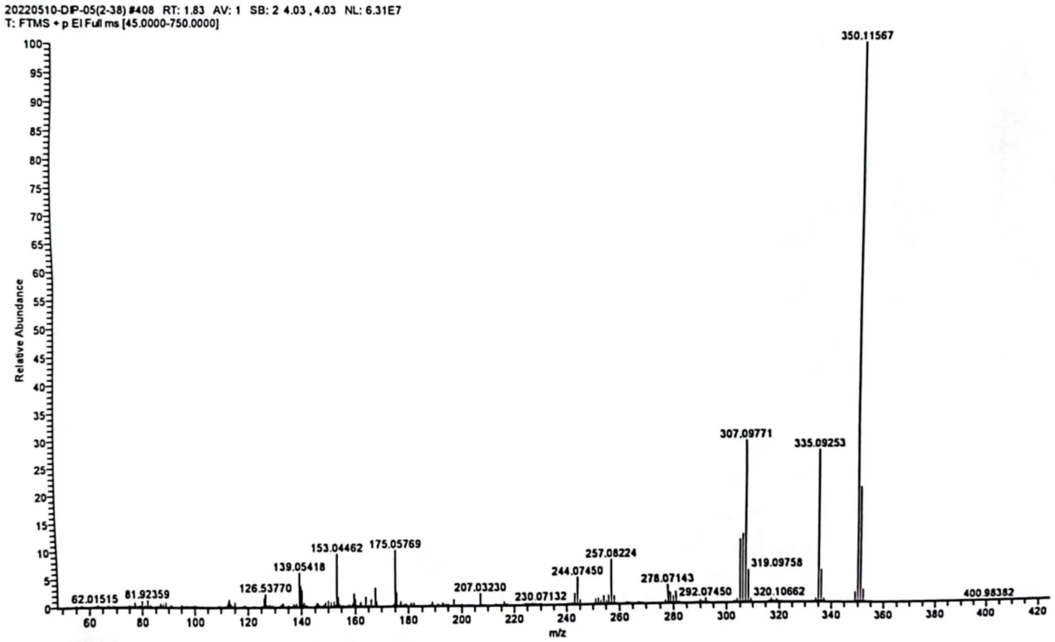

Supplementary Figure 11. HRMS spectrum of **4**.

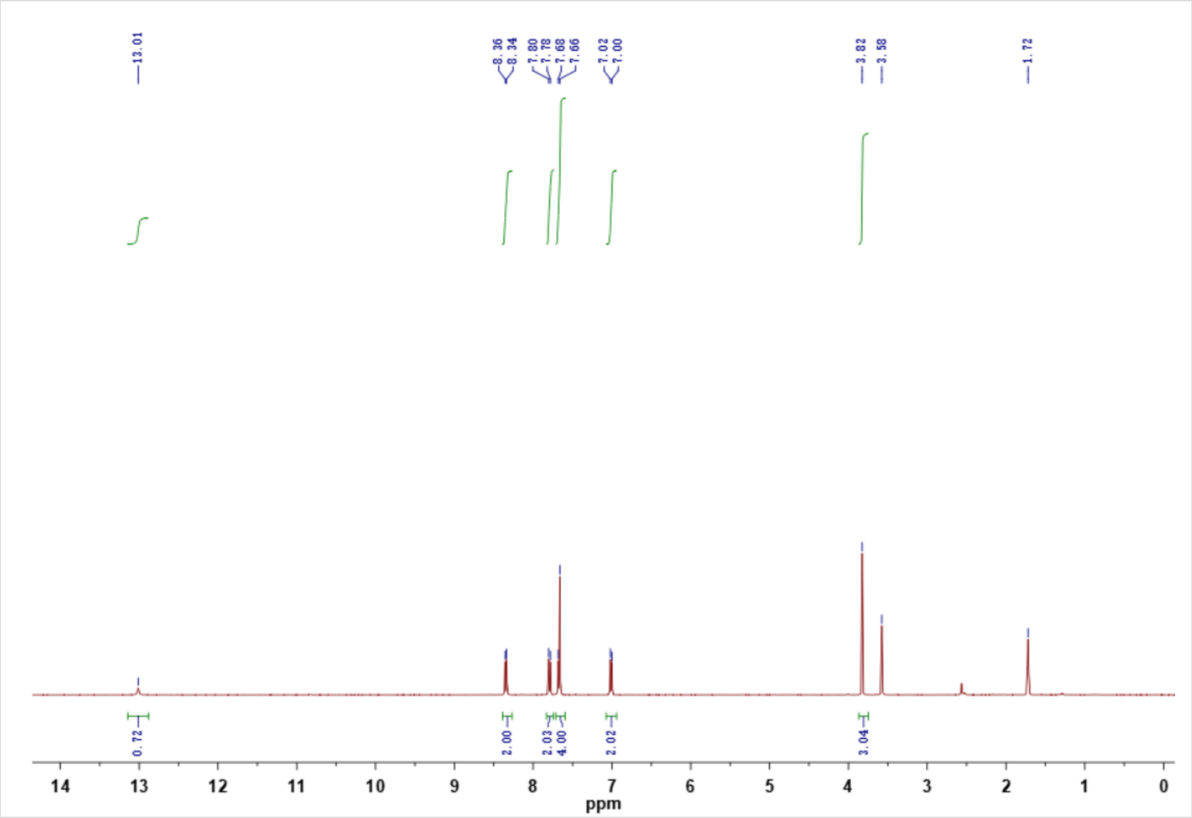

Supplementary Figure 12. ^{1}H NMR spectrum of **4** in THF-d8.

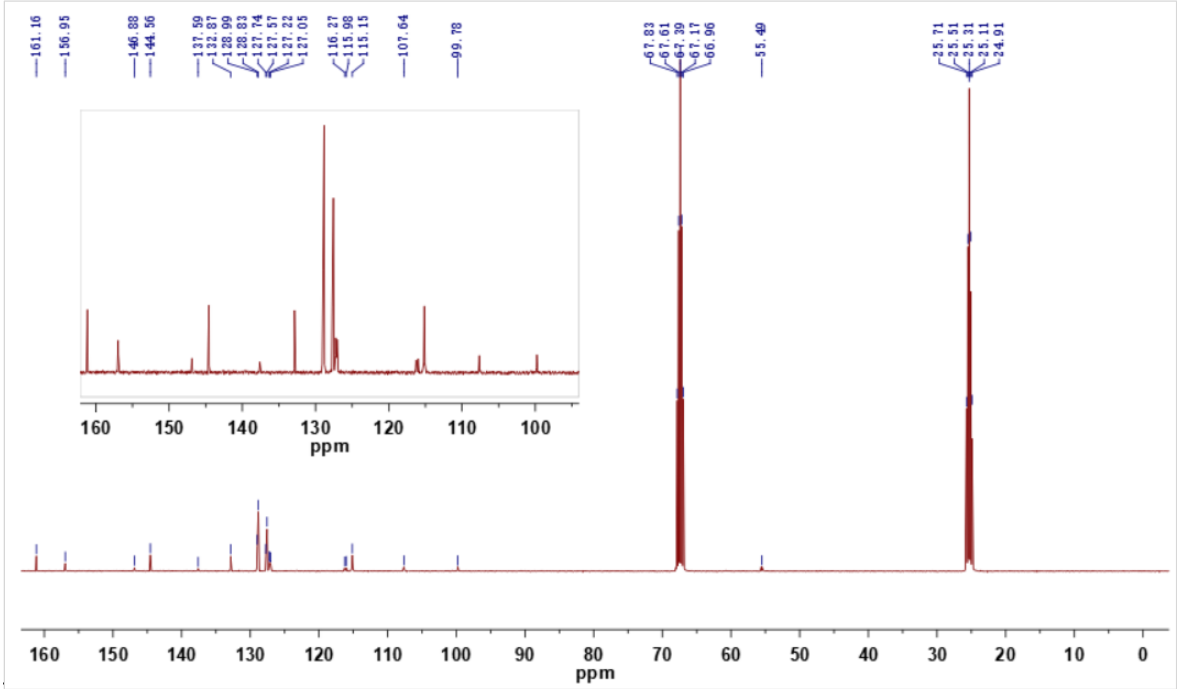

Supplementary Figure 13. ^{13}C NMR spectrum of **4** in THF-d8.

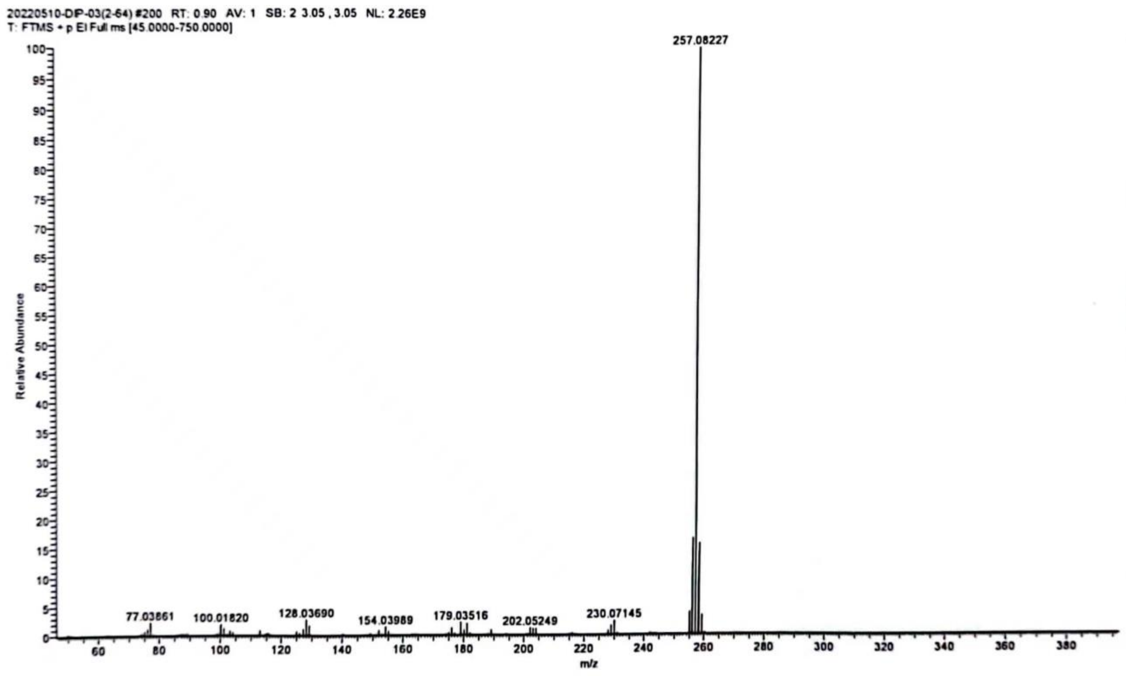

Supplementary Figure 14. HRMS spectrum of **6.**

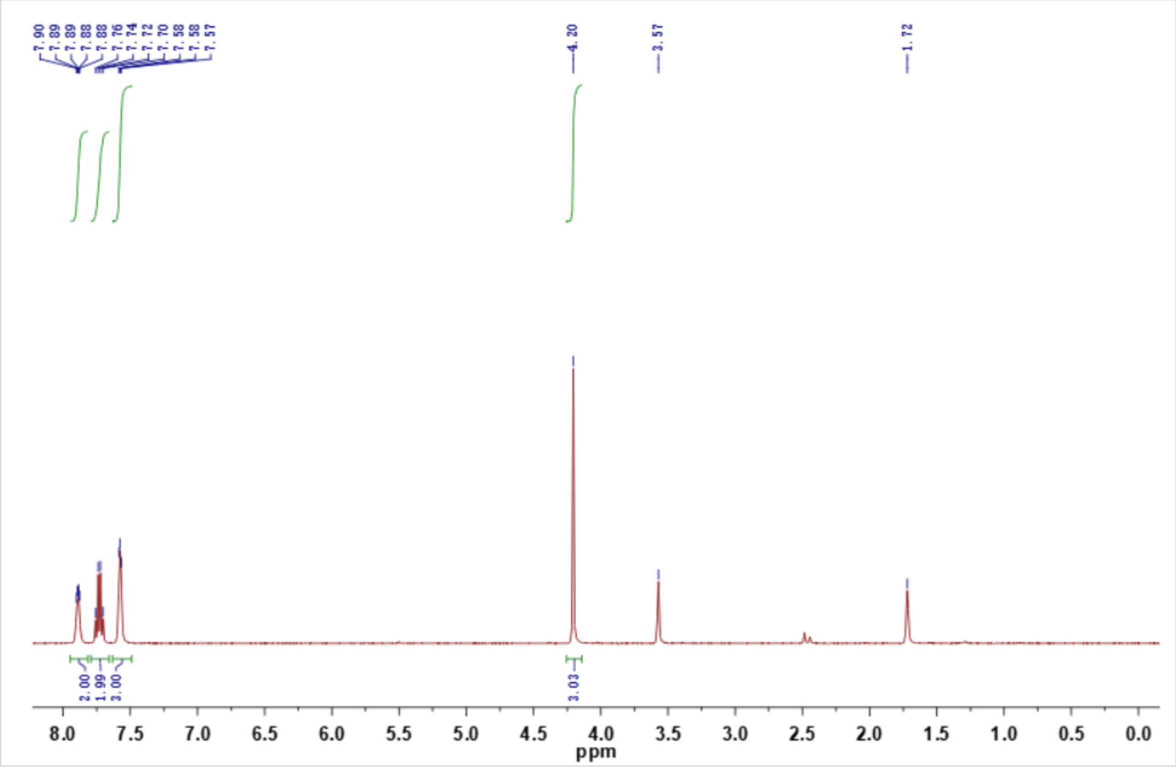

Supplementary Figure 15. ^{1}H NMR spectrum of **6** in THF-d8.

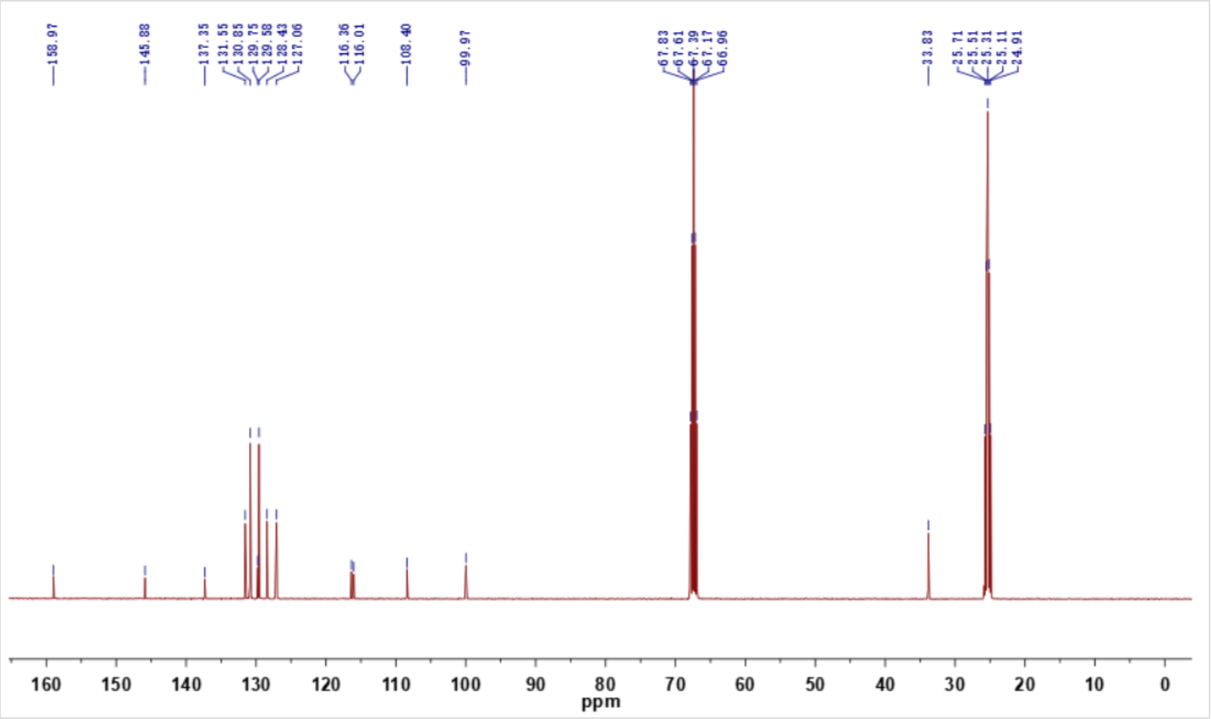

Supplementary Figure 16. ^{13}C NMR spectrum of **6** in THF-d8.

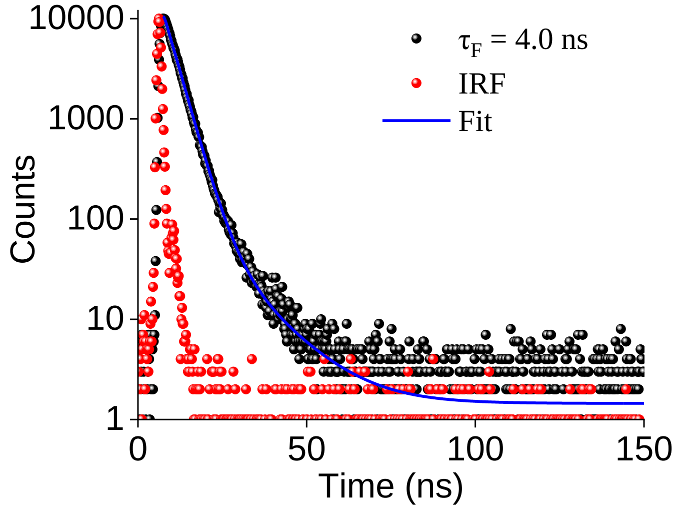

Supplementary Figure 17. Fluorescence lifetime decay curve of 0.1 wt% **1**@PA6 film at 320 nm. IRF: instrument response function.

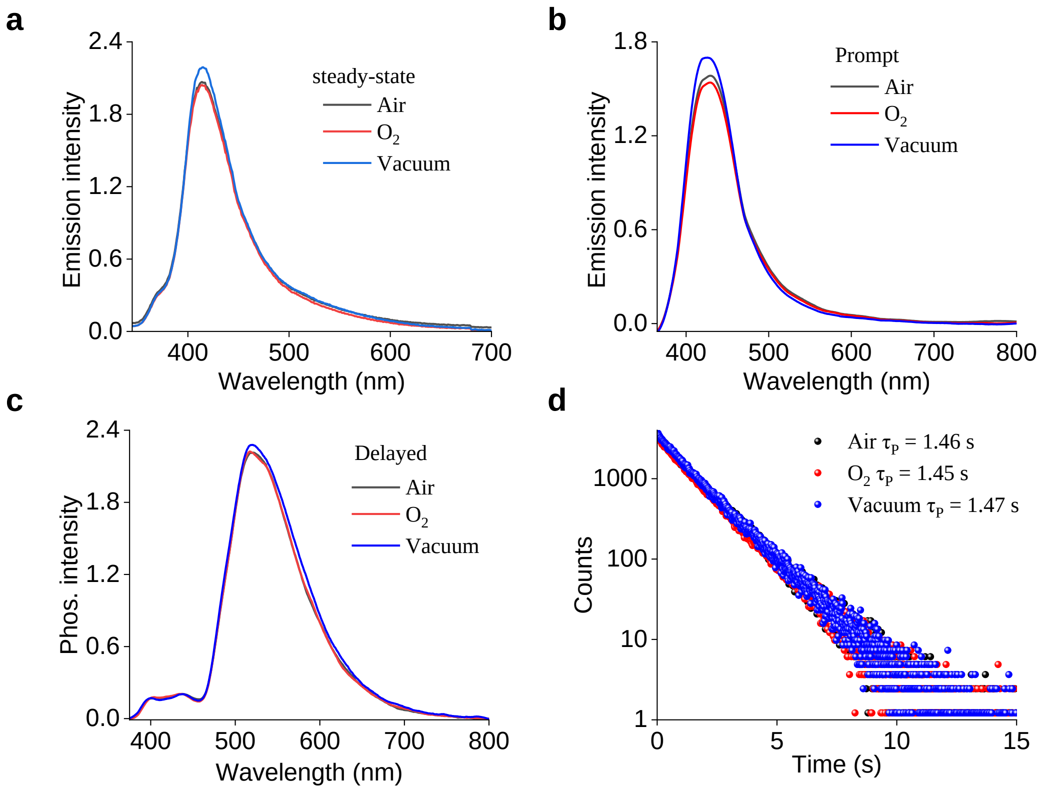

Supplementary Figure 18. (a) Steady-state, (b) prompt, and (c) delayed photoluminescence spectra and (d) lifetime decay curves of the phosphorescence emission band at 510 nm of 0.1 wt% **1**@PA6 measured in air, under vacuum, or after being exposed to oxygen for 30 min. The steady-state emissions are obtained with a xenon lamp (450 W). The prompt and delayed emissions are obtained with a microsecond flash lamp (100 W) without or with time delay (5 ms), respectively.

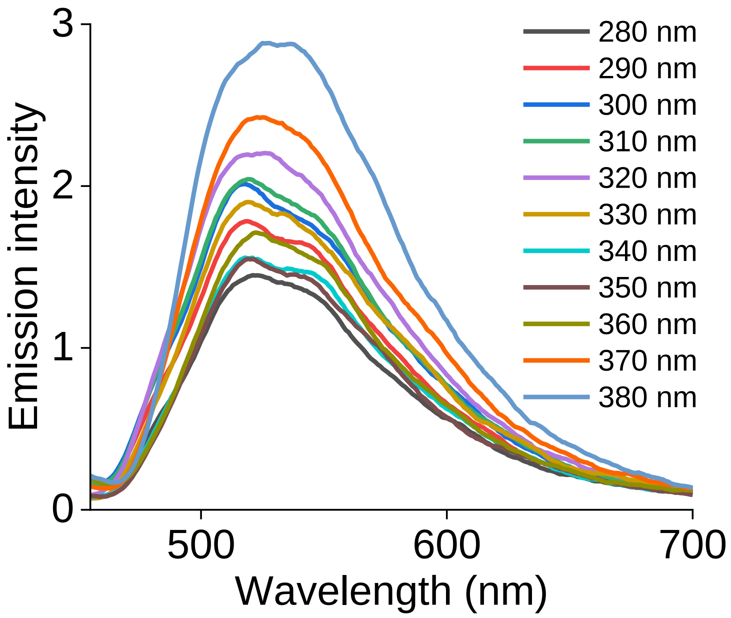

Supplementary Figure 19. Delayed emission spectra of 0.1wt% **1**@PA6 film excited at different wavelength from 280 to 380 nm with a delay time of 5 ms.

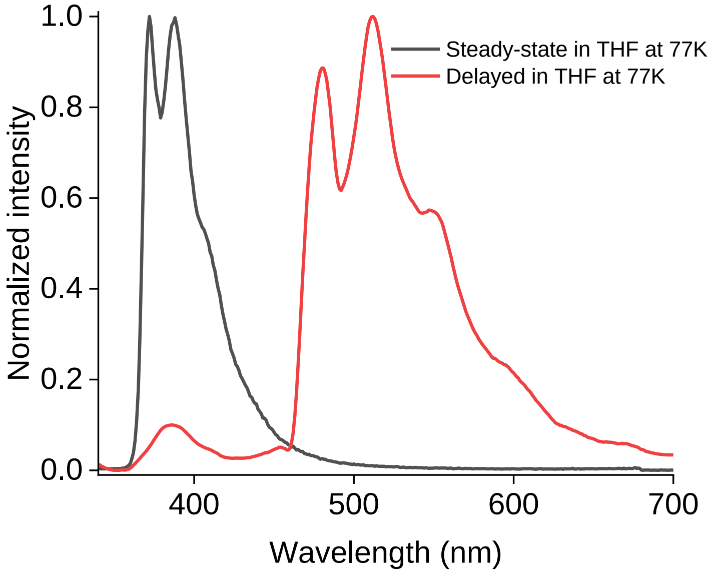

Supplementary Figure 20. Steady-state fluorescence and delayed emission spectra of **1** in THF (5×10^{-5} M) at 77 K with a delay time of 5 ms.

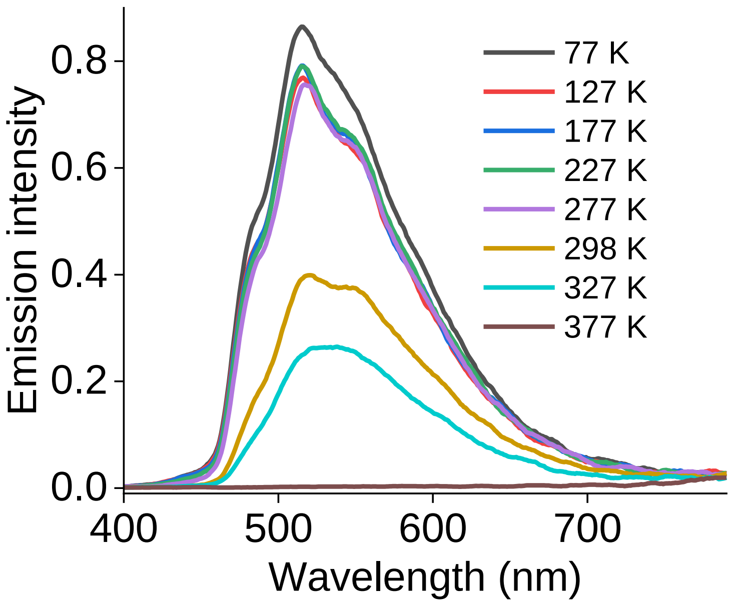

Supplementary Figure 21. Temperature-dependent phosphorescence spectra of 0.1 wt% **1**@PA6 film from 77 to 377 K. Excited at 320 nm. Delay time: 5 ms.

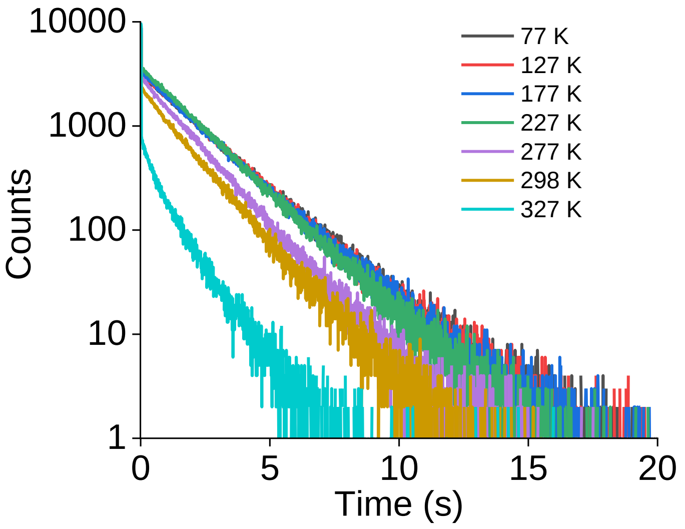

Supplementary Figure 22. Temperature-dependent phosphorescence lifetime decay curves of 0.1 wt% **1**@PA6 film monitored at 510 nm under kinetics measurements (Xenon light source, excited at 320 nm).

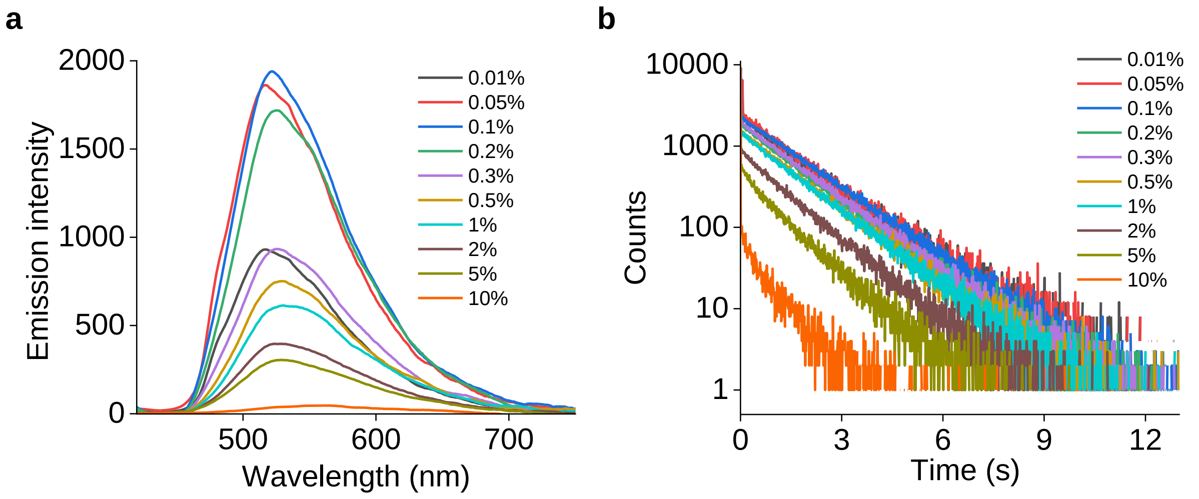

Supplementary Figure 23. (a) Phosphorescence spectra excited at 320 nm and (b) lifetime decay curves of phosphorescence emission band at 510 nm of **1**@PA6 film with different doping concentrations of **1**.

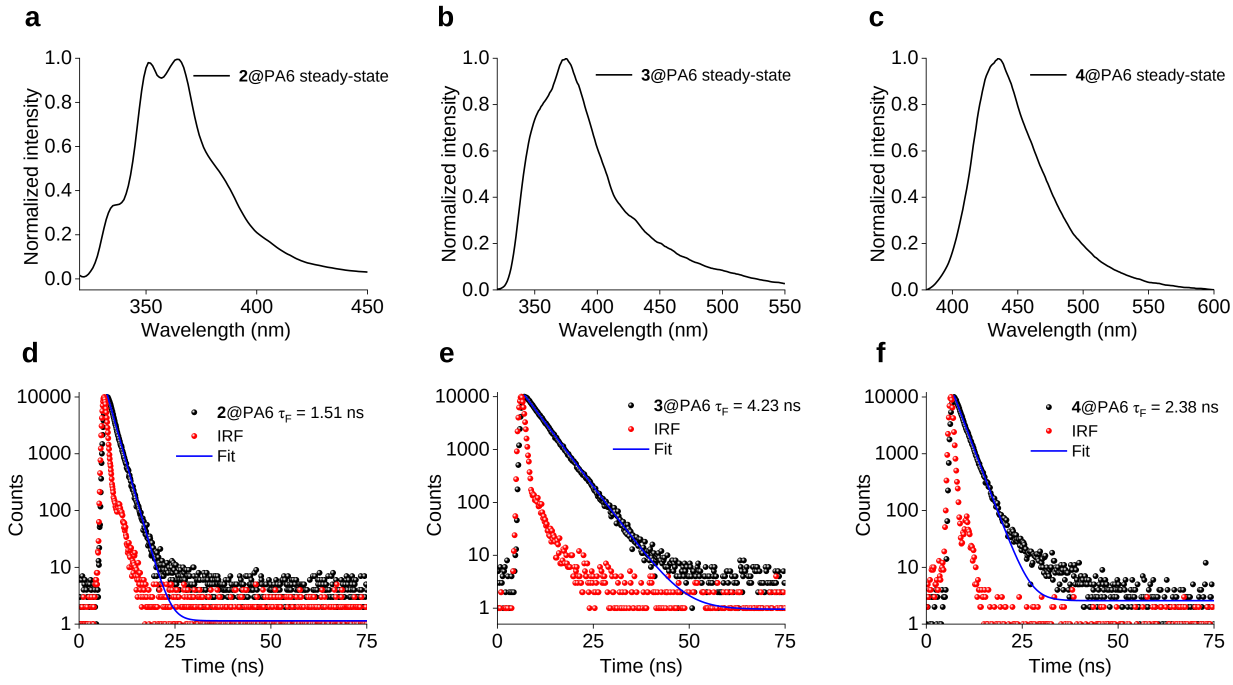

Supplementary Figure 24. (a, b, c) steady-state emission and (d, e, f) lifetime decay curves of fluorescence emission of (a, d) **2**@PA6, (b, e) **3**@PA6, and (c, f) **4**@PA6 film under ambient conditions at rt (0.1 wt% doping ratio for all).

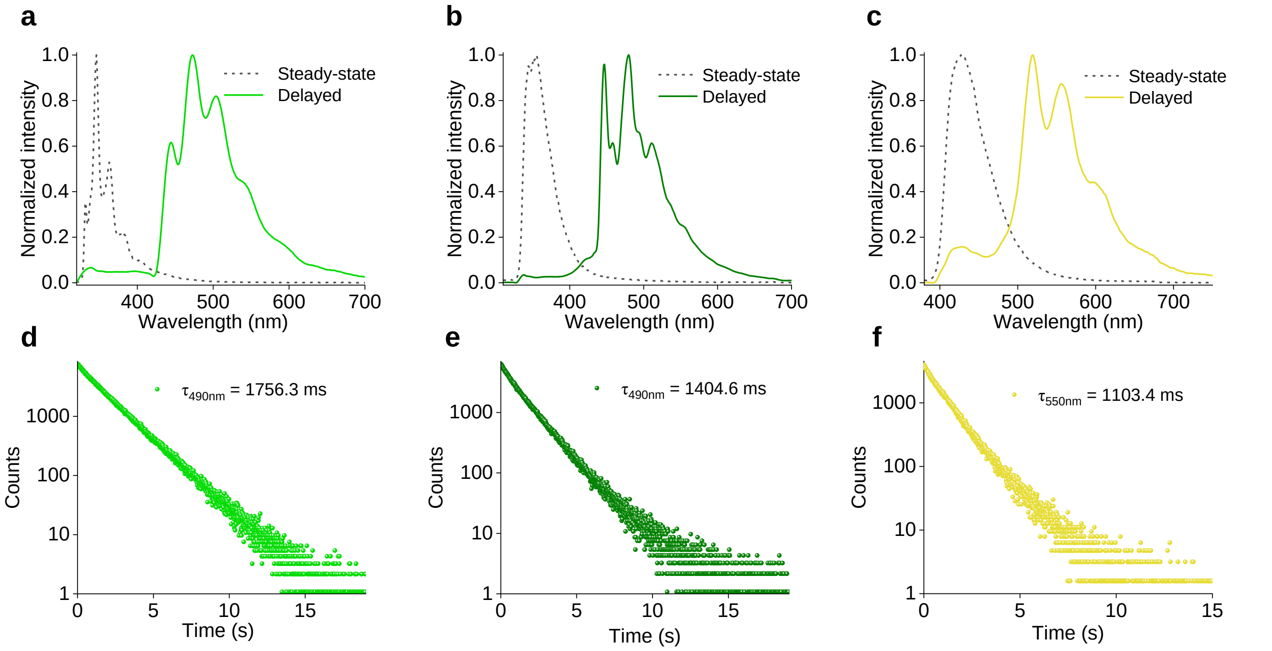

Supplementary Figure 25. (a, b, c) Steady-state fluorescence and delayed emission spectra and (d, e, f) decay profiles of the long-lived emissions of (a, d) **2** at 470 nm, (b, e) **3** at 480 nm, and (c, f) **4** at 520 nm in THF (5×10^{-5} M) at 77 K.

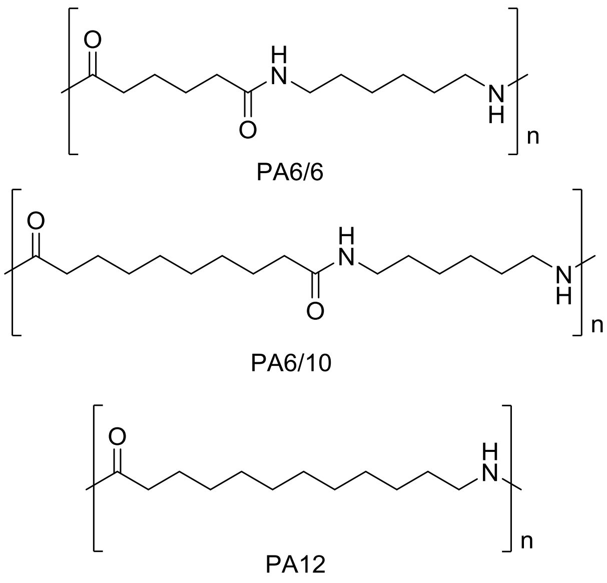

Supplementary Figure 26. The chemical structures of PA6/6, PA6/10 and PA12.

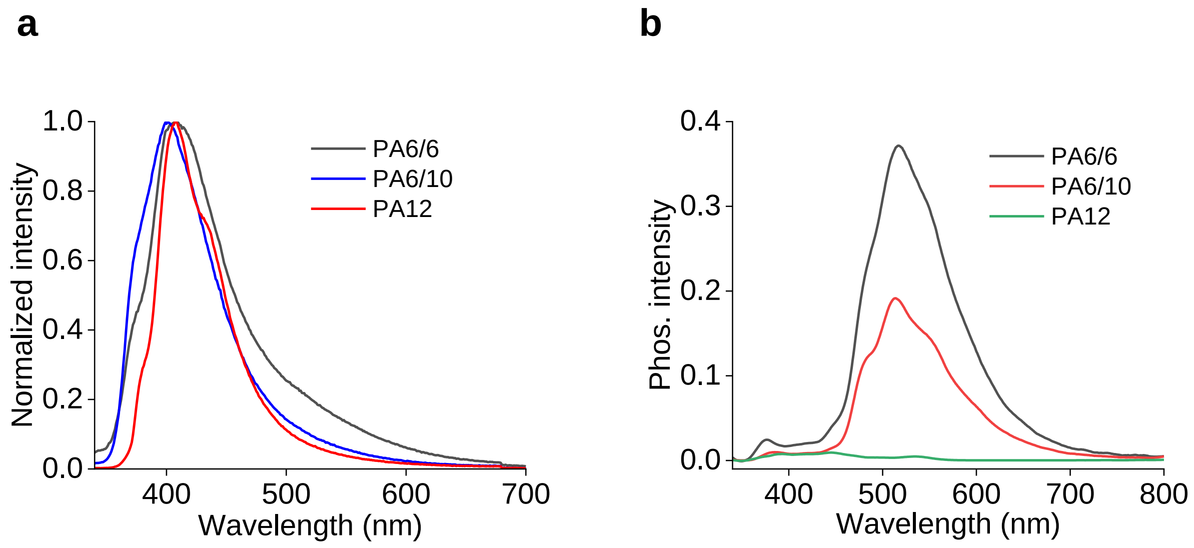

Supplementary Figure 27. (a) Steady-state fluorescence and (b) delayed emission spectra of PA6/6, PA6/10, and PA12 films doped with 0.1 wt% of compound **1**.

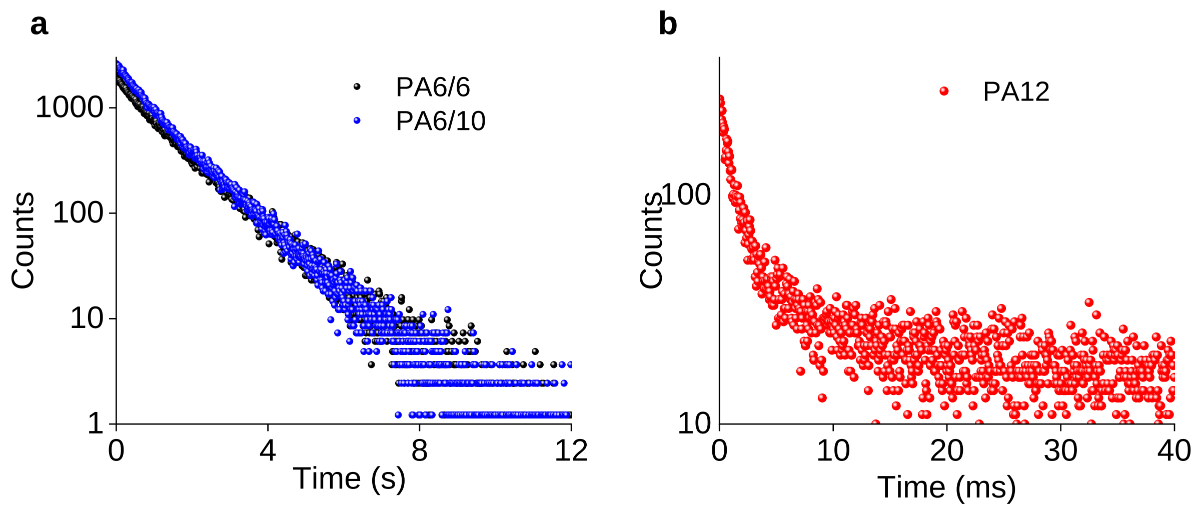

Supplementary Figure 28. Phosphorescence decay curves at 510 nm of (a) PA6/6 and PA6/10 and (b) PA12 films doped with 0.1 wt% of compound **1**.

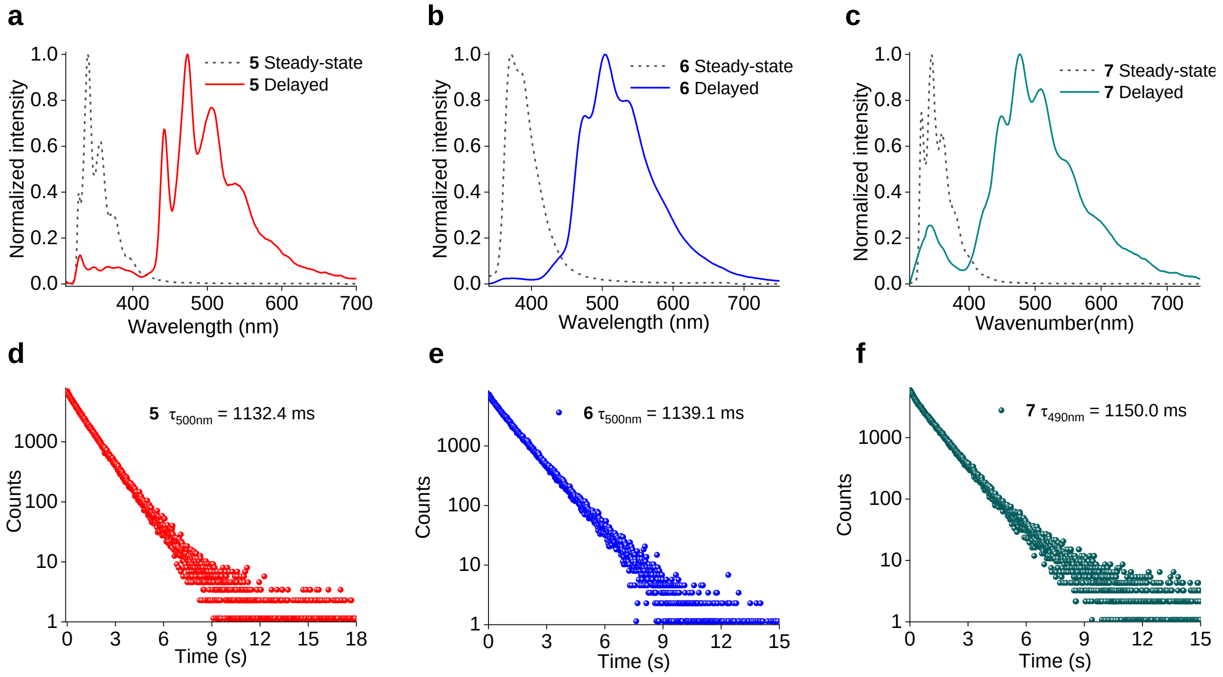

Supplementary Figure 29. (a, b, c) Steady-state fluorescence and delayed emission spectra and (d, e, f) decay profiles of the long-lived emissions of (a, d) **5** at 470 nm, (b, e) **6** at 510 nm, and (c, f) **7** at 480 nm in THF (5×10^{-5} M) at 77 K.

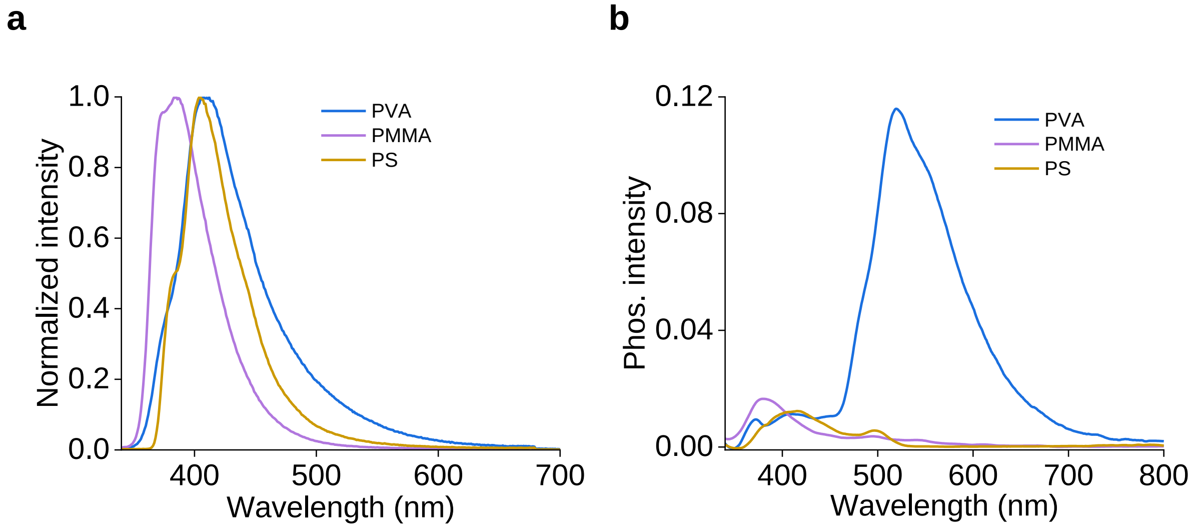

Supplementary Figure 30. (a) Steady-state fluorescence and (b) delayed emission spectra of PVA, PMMA, and PS polymers doped with 0.1 wt% of compound **1**.

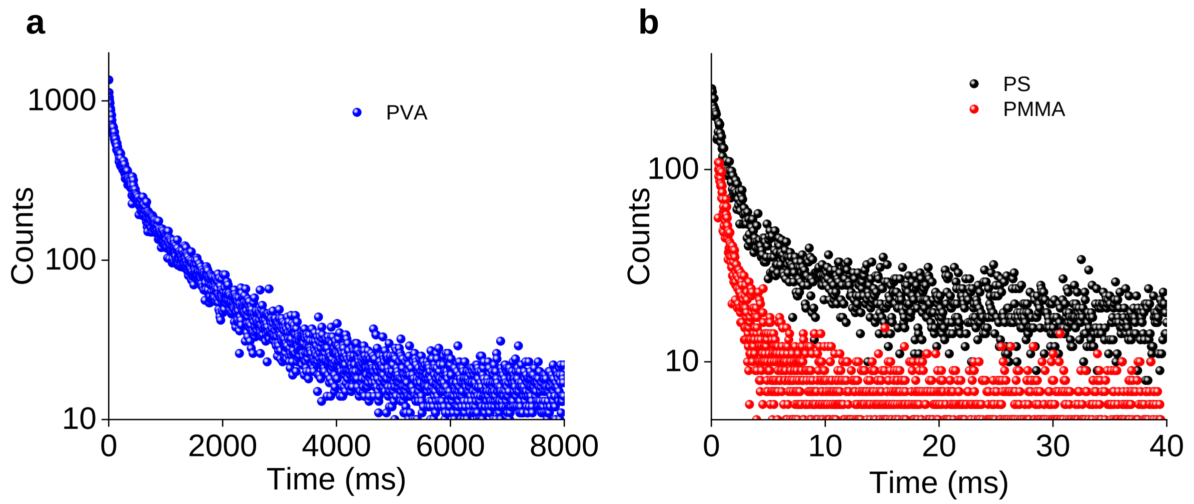

Supplementary Figure 31. Phosphorescence decay curves at 510 nm of (a) PVA and (b) PS and PMMA polymers doped with 0.1 wt% of compound **1**.

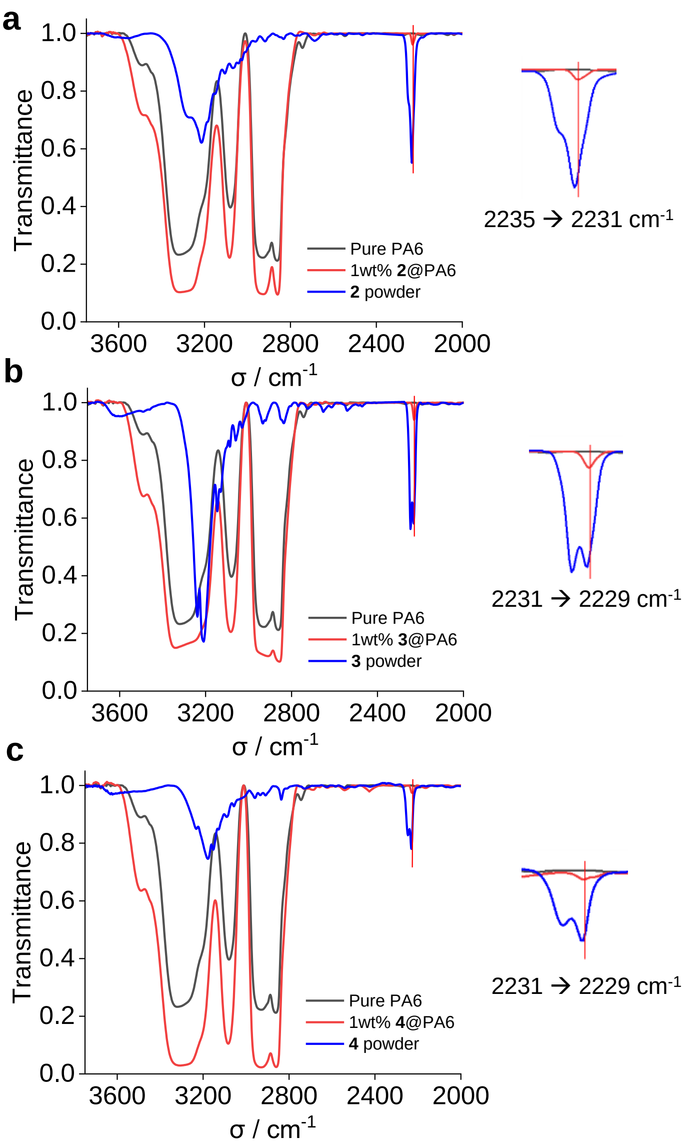

Supplementary Figure 32. FTIR spectra of (a) **2**@PA6, (b) **3**@PA6, and (c) **4**@PA6 in comparison with those of the powder of pure **2** – **4** and pure PA6.

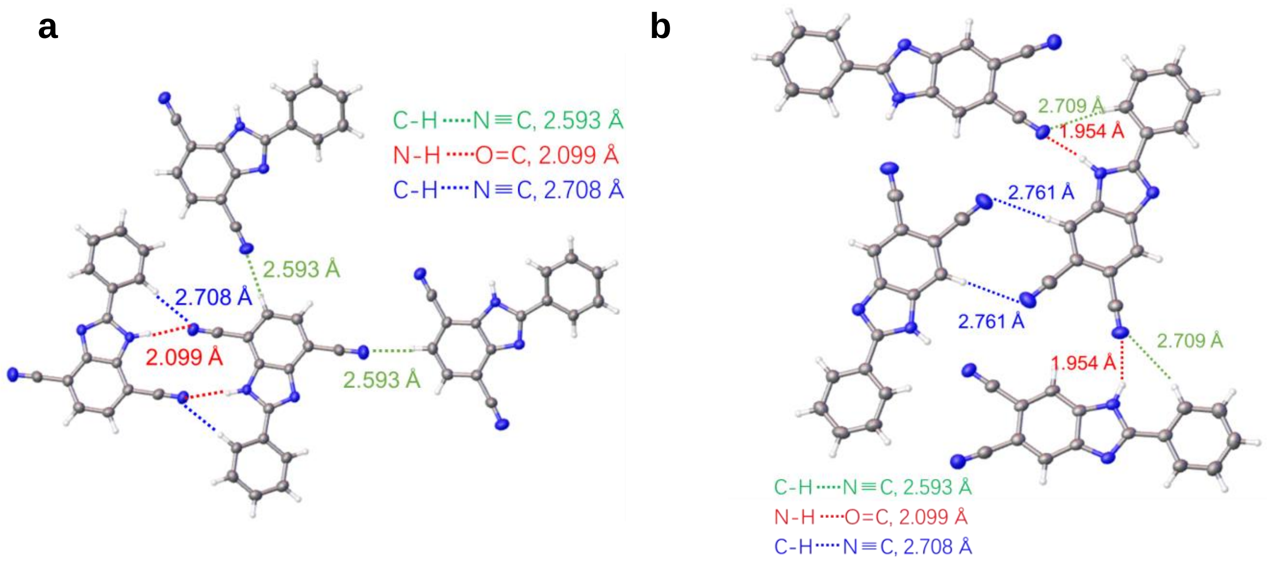

Supplementary Figure 33. Single-crystal molecular packing of (a) **1** and (b) **2** showing the presence of hydrogen bondings.

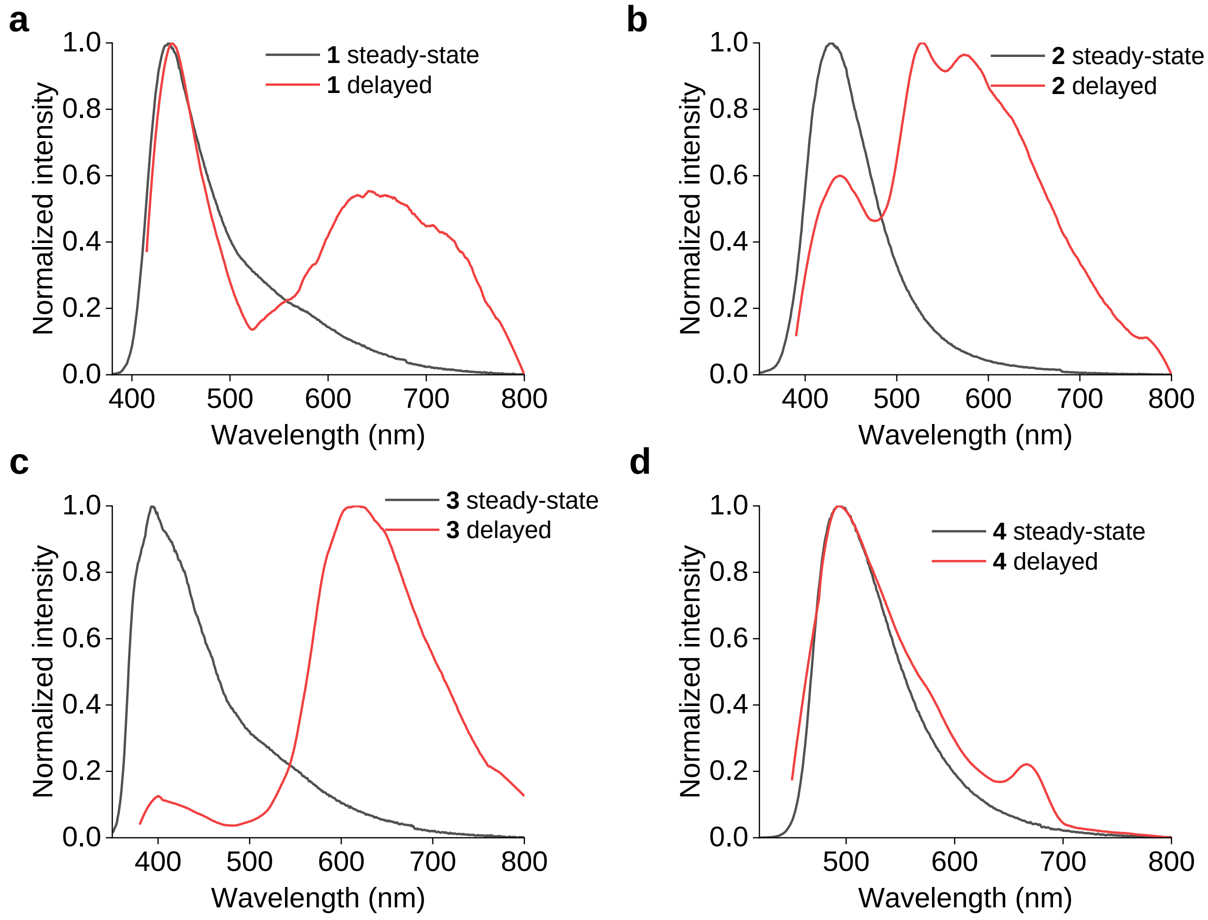

Supplementary Figure 34. Normalized steady-state and delayed emission spectra of the crystalline powder of (a) **1**, (b) **2**, (c) **3**, and (d) **4** under ambient conditions. Delayed time: 0.5 ms.

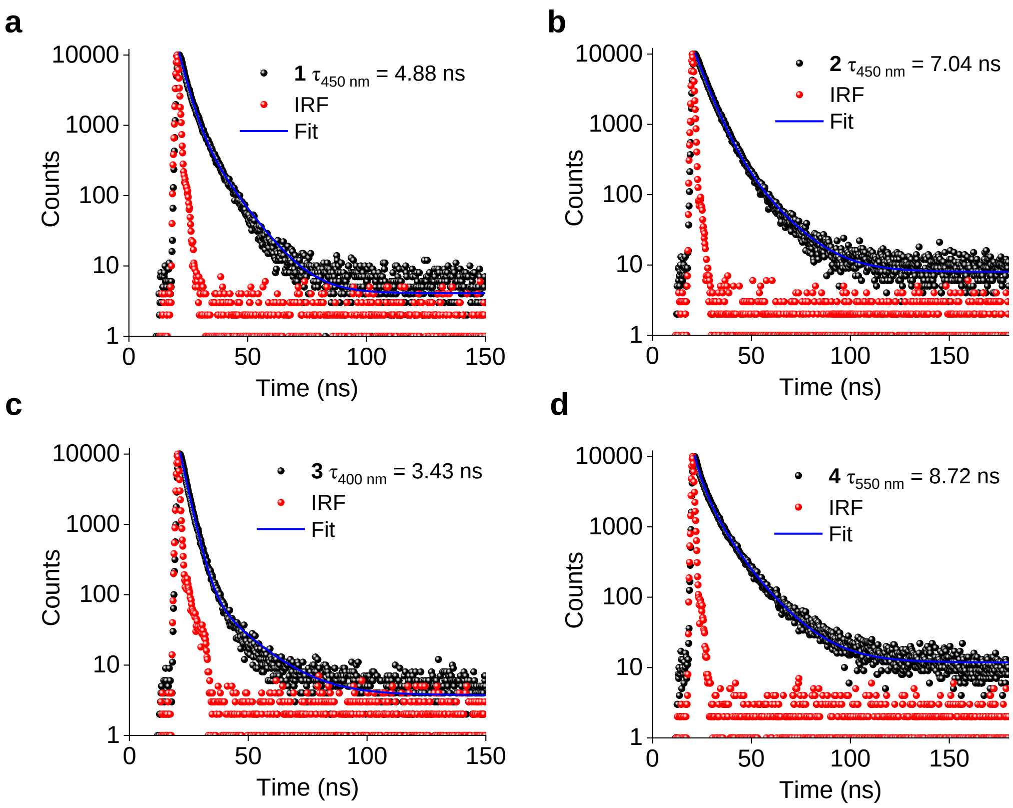

Supplementary Figure 35. Lifetime decay curves of the fluorescence emission of the crystalline powder of (a) **1**, (b) **2**, (c) **3**, and (d) **4** under ambient conditions.

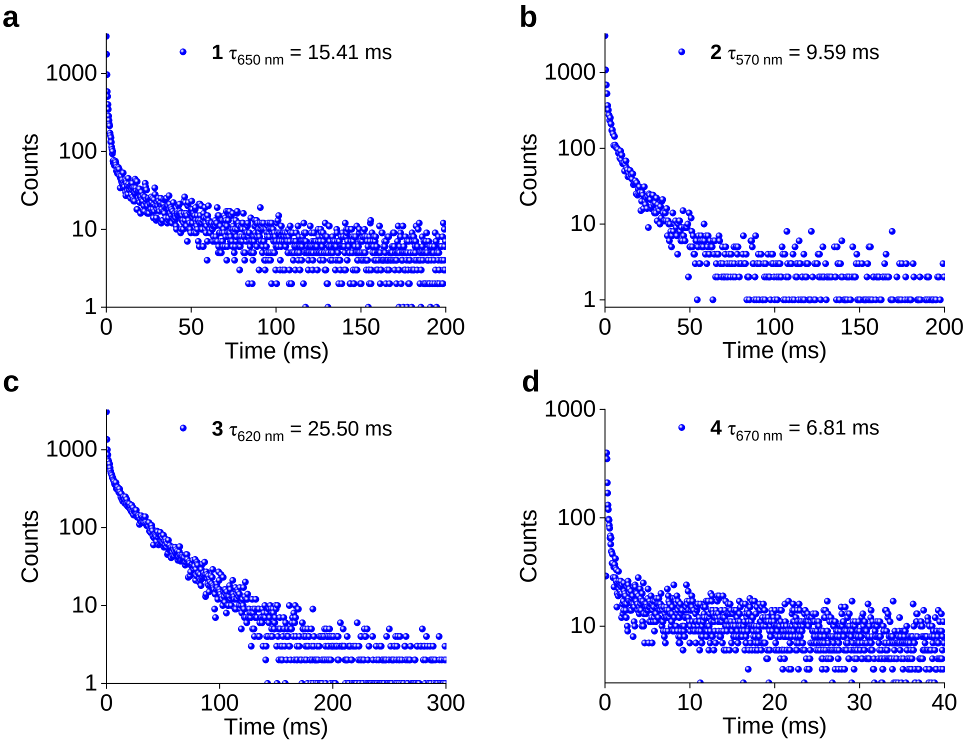

Supplementary Figure 36. Lifetime decay curves of the phosphoresce emission the crystalline powder of (a) **1**, (b) **2**, (c) **3**, and (d) **4** under ambient conditions. Measured after 0.5 ms of delay.

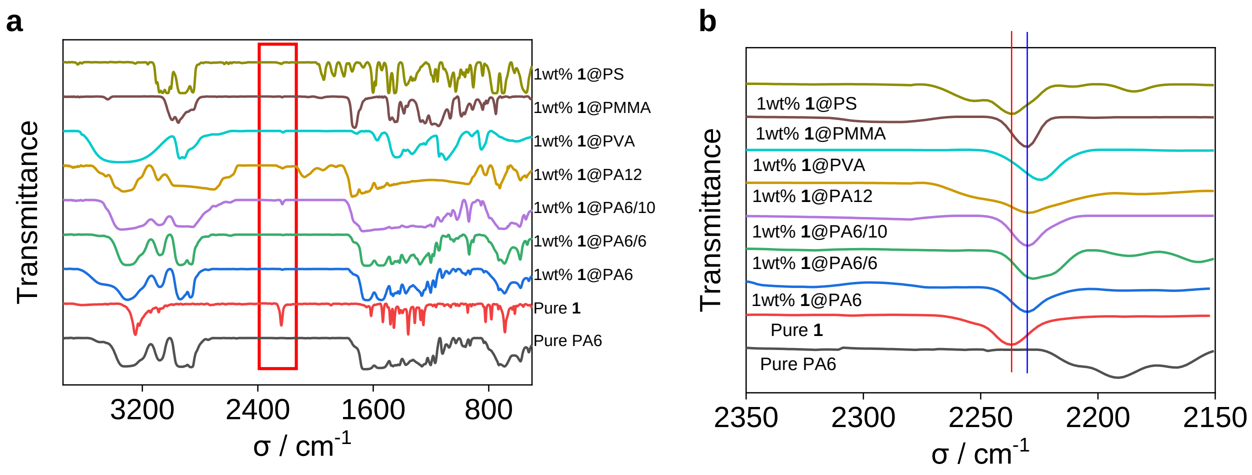

Supplementary Figure 37. FTIR spectra of **1** in different hosts.

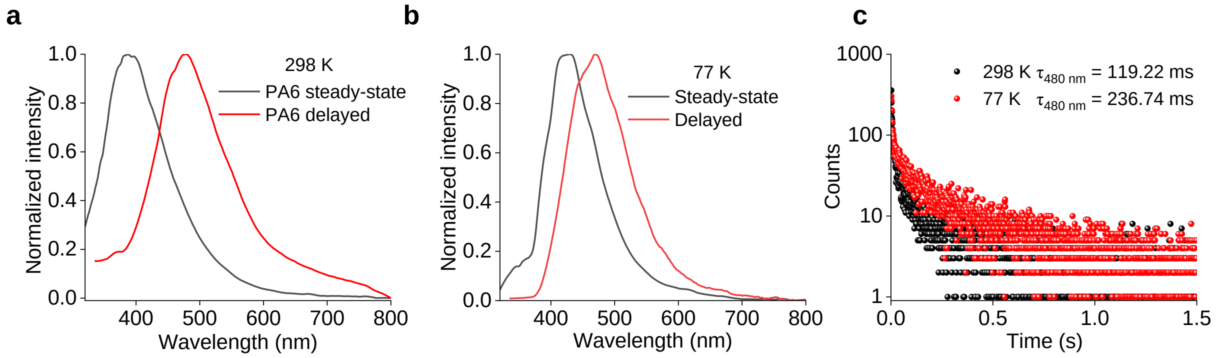

Supplementary Figure 38. (a,b) Normalized steady-state and delayed emission spectra of pure PA6 film at (a) 298 K and (b) 77 K. (c) Phosphorescence decay curves of PA6 at 480 nm at 298 K and 77 K.

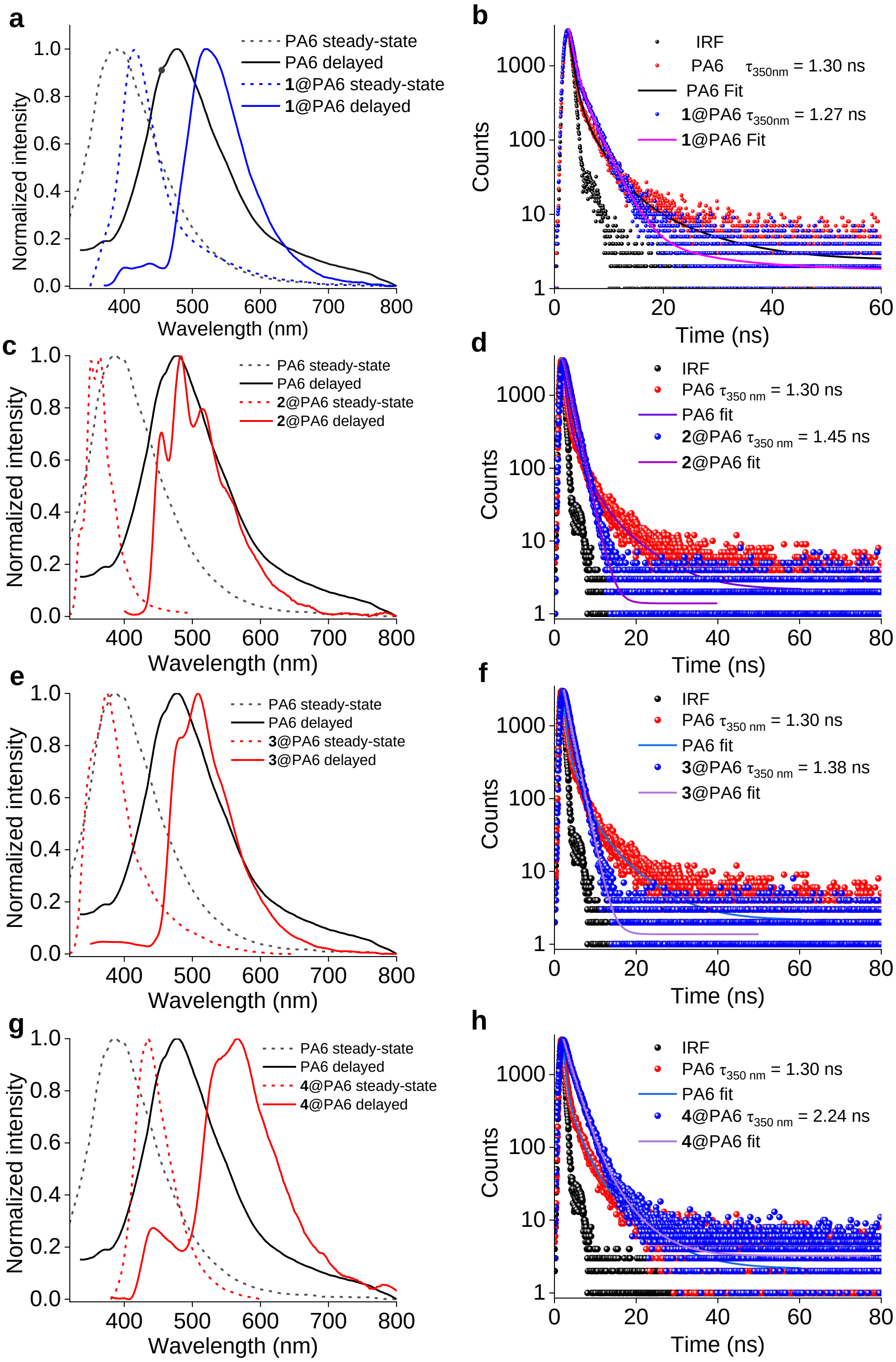

Supplementary Figure 39. (a,c,e,g) Steady-state and delayed emission spectra of PA6, **1**@PA6, **2**@PA6, **3**@PA6, and **4**@PA6. (b,d,f,h) Fluorescence decays and fitted lifetimes at 350 nm of PA6, **1**@PA6, **2**@PA6, **3**@PA6, and **4**@PA6 (excited at 290 nm).

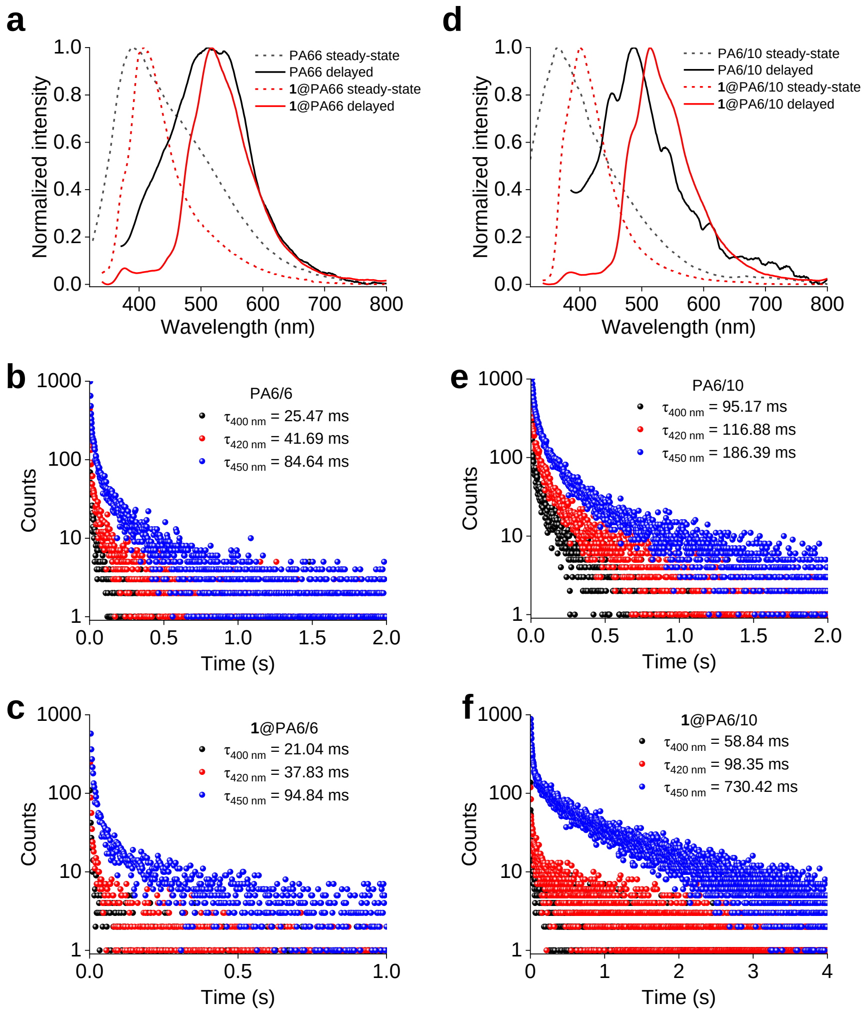

Supplementary Figure 40. (a) Steady-state and delayed emission spectra of PA6/6 and **1**@PA6/6. (b,c) Phosphorescence decays and fitted lifetimes at indicated wavelength of (b) PA6/6 and (c) **1**@PA6/6 (excited at 290 nm). (d) Steady-state and delayed emission spectra of PA6/10 and **1**@PA6/10. (e,f) Phosphorescence decays and fitted lifetimes at indicated wavelength of (e) PA6/10 and (f) **1**@PA6/10 (excited at 290 nm).

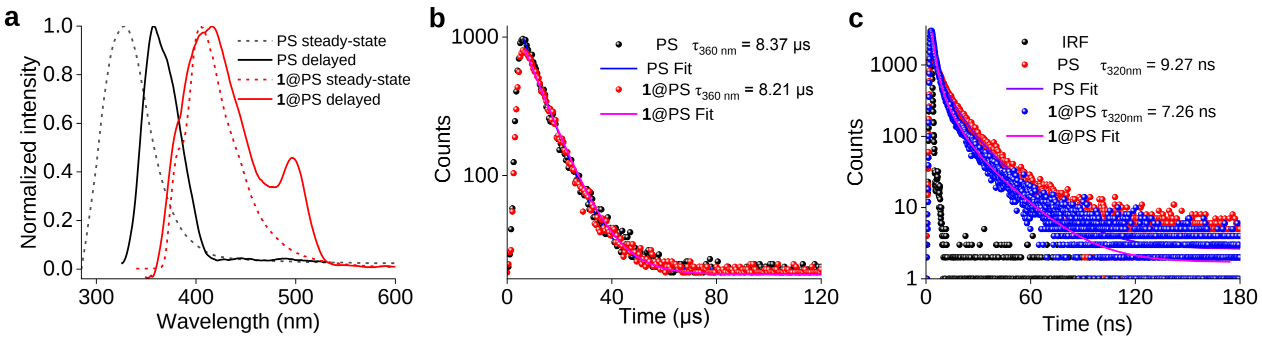

Supplementary Figure 41. (a) Steady-state and phosphorescence spectra of PS and **1**@PS. (b) Phosphorescence decays and fitted lifetimes at 360 nm of PS and **1**@PS (excited at 290 nm). (c) Fluorescence decays and fitted lifetimes at 320 nm of PS and **1**@PS. The phosphorescence of **1**@PS is rather weak. The below-zero values of **1**@PS in panel (a) are caused by the instrument's background noise.

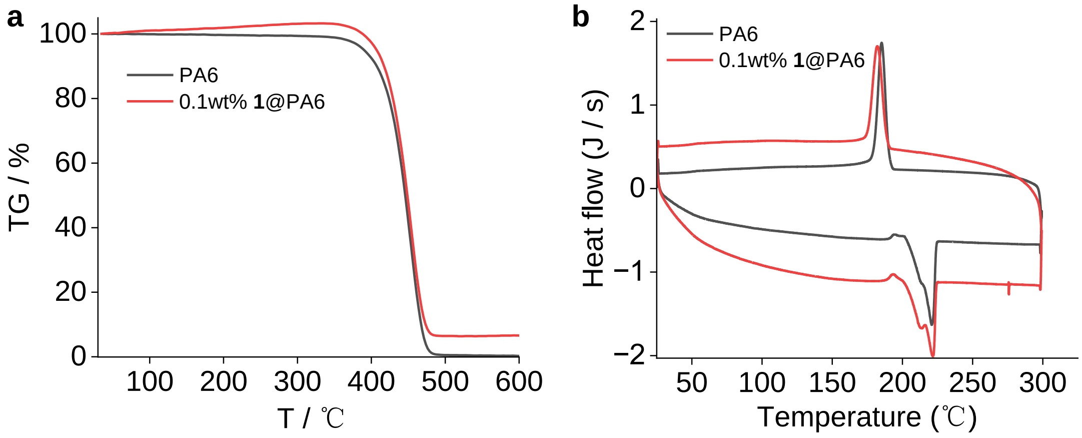

Supplementary Figure 42. (a) TGA and (b) DSC curves of pure PA6 film and 0.1 wt% **1**@PA6 film.

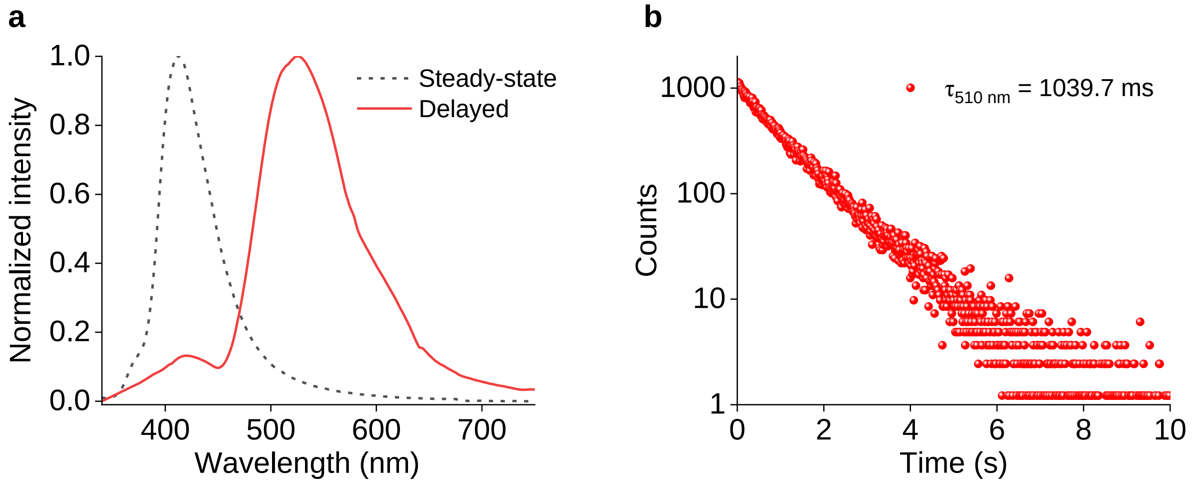

Supplementary Figure 43. (a) Normalized steady-state and delayed emission and (b) lifetime decay curves of phosphorescence emission band at 510 nm of 0.1 wt% **1**@PA6 fiber.

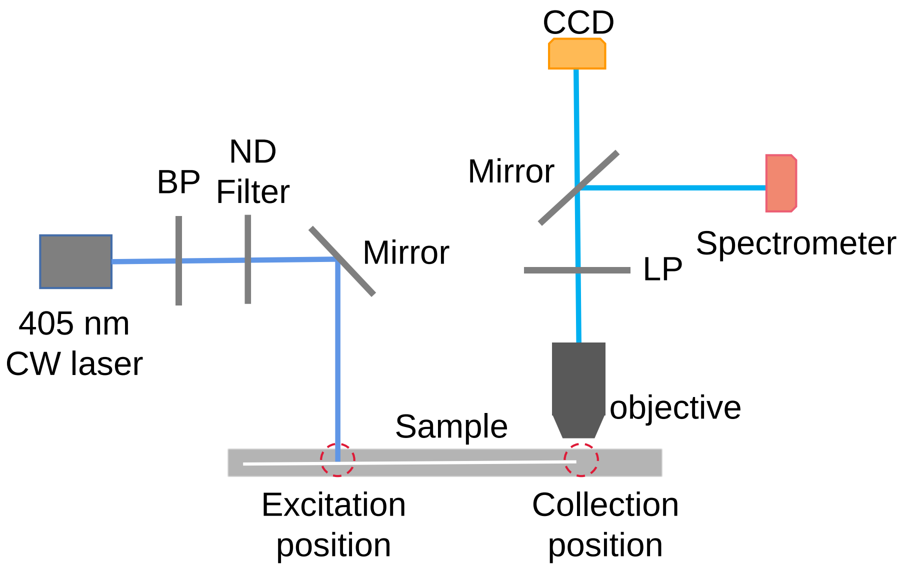

Supplementary Figure 44. Schematic demonstration of the experimental setups for optical waveguide studies

Supplementary Tables

Supplementary Table 1. Phosphorescence lifetime (τ_P) and phosphorescence quantum yield (Φ_P) of PA6 films doped with different amount (wt%) of compound **1**.

Ratio (wt%)	τ_P (s)	Φ_P (%)
0.01	1.42	30.6
0.05	1.44	35.3
0.1	1.51	48.3
0.2	1.42	33.6
0.3	1.39	27.5
0.5	1.37	16.1
1	1.36	6.46
2	1.21	3.21
5	1.03	1.07
10	0.665	~

Supplementary Table 2. Crystallographic data of single crystal of **1** and **2**.

Compound	**1**	**2**
CCDC number	2337657	2337687
Empirical formula	$C_{15}H_8N_4$	$C_{15}H_8N_4$
Formula weight	244.26	244.26
Temperature (K)	169.99(10)	169.99(13)
Crystal system	monoclinic	monoclinic
Space group	$P2_1/n$	C2/c
a (Å)	3.8311(2)	25.5374(3)
b (Å)	21.9130(11)	11.73420(11)
c (Å)	14.0092(6)	32.4465(3)
α (°)	90	90
β (°)	92.083(4)	104.6962(11)
γ (°)	90	90
Volume ($Å^3$)	1175.31(10)	9404.86(17)
Z	4	8
Density (g/cm^3)	1.3803	1.3799
R1 (final)	0.1230	0.0358
wR2 (final)	0.3247	0.0927
R1 (all)	0.1296	0.0412
wR2 (all)	0.3302	0.0960
