# Supplementary material for: Nylons with Highly-Bright and Ultralong Organic Room-Temperature Phosphorescence
Source: Nat Commun. 2024 May 23;15:4402. doi: 10.1038/s41467-024-48836-7 (PMC11116439; doi:10.1038/s41467-024-48836-7)
Supplement: Supplementary file 3 — Description of Additional Supplementary Files [file 41467_2024_48836_MOESM3_ESM.pdf]

Description of Additional Supplementary Files

File Name: Supplementary Data 1
Description: Cartesian coordinates of compound 1.

File Name: Supplementary Movie 1
Description: The movie of 0.1 wt% 1@PA6 film showing long-persistent phosphorescence before and after turning off the UV lamp 365 nm irradiation (MP4).

File Name: Supplementary Movie 2
Description: The movie of 0.1 wt% 1@PA6 fibers showing long-persistent phosphorescence before and after turning off the UV lamp 365 nm irradiation (MP4)
